# The Food Poisoning Toxins of *Bacillus cereus*

**DOI:** 10.3390/toxins13020098

**Published:** 2021-01-28

**Authors:** Richard Dietrich, Nadja Jessberger, Monika Ehling-Schulz, Erwin Märtlbauer, Per Einar Granum

**Affiliations:** 1 Department of Veterinary Sciences, Faculty of Veterinary Medicine, Ludwig Maximilian University of Munich, Schönleutnerstr. 8, 85764 Oberschleißheim, Germany; r.dietrich@mh.vetmed.uni-muenchen.de (R.D.); e.maertlbauer@mh.vetmed.uni-muenchen.de (E.M.); 2 Department of Pathobiology, Functional Microbiology, Institute of Microbiology, University of Veterinary Medicine Vienna, 1210 Vienna, Austria; Monika.Ehling-Schulz@vetmeduni.ac.at; 3 Department of Food Safety and Infection Biology, Faculty of Veterinary Medicine, Norwegian University of Life Sciences, P.O. Box 5003 NMBU, 1432 Ås, Norway; per.einar@gmico.no

**Keywords:** *Bacillus cereus*, hemolysin BL, non-hemolytic enterotoxin, cytotoxin K, cereulide, pore formation, cytotoxicity, food poisoning

## Abstract

*Bacillus cereus* is a ubiquitous soil bacterium responsible for two types of food-associated gastrointestinal diseases. While the emetic type, a food intoxication, manifests in nausea and vomiting, food infections with enteropathogenic strains cause diarrhea and abdominal pain. Causative toxins are the cyclic dodecadepsipeptide cereulide, and the proteinaceous enterotoxins hemolysin BL (Hbl), nonhemolytic enterotoxin (Nhe) and cytotoxin K (CytK), respectively. This review covers the current knowledge on distribution and genetic organization of the toxin genes, as well as mechanisms of enterotoxin gene regulation and toxin secretion. In this context, the exceptionally high variability of toxin production between single strains is highlighted. In addition, the mode of action of the pore-forming enterotoxins and their effect on target cells is described in detail. The main focus of this review are the two tripartite enterotoxin complexes Hbl and Nhe, but the latest findings on cereulide and CytK are also presented, as well as methods for toxin detection, and the contribution of further putative virulence factors to the diarrheal disease.

## 1. Introduction

*Bacillus cereus* is estimated to be responsible for 1.4%–12% of all food poisoning outbreaks worldwide [1]. In the European Union, bacterial toxins (*Clostridium*, *Staphylococcus* and *B. cereus*) accounted for 17.7% (2016) and 15.9% (2017) of all registered food- and water-borne outbreaks, which ranked them second behind *Salmonella* [2,3]. With 98 registered outbreaks in the EU in 2018, *B. cereus* toxins ranked in fifth place behind *Salmonella*, *Campylobacter*, the norovirus and *Staphylococcus* toxins. Among these was also one large food poisoning outbreak with more than 100 affected persons. Furthermore, six fatal cases were attributed to bacterial toxins (*Clostridium botulinum*, *Clostridium perfringens* and *B. cereus*) [4].

Basically, *B. cereus* is responsible for two types of gastrointestinal diseases. The emetic kind of illness is mainly characterized by nausea and emesis, which appear as soon as half an hour after consumption of the contaminated food and are clinically indistinguishable from intoxications with *Staphylococcus aureus* enterotoxins [5]. In this classical food intoxication, the emetic toxin cereulide is pre-formed during vegetative growth of *B. cereus* in foodstuffs and the consumption of the bacteria is not necessary [6]. Indeed, there are several reports of outbreaks where only the cereulide toxin was detected in the food, but no bacteria could be isolated [7]. Nevertheless, it is generally thought that at least 10^3^–10^5^ *B. cereus* per g food are needed to produce cereulide in disease-provoking concentrations [5,6,7,8,9]. Cereulide is a cyclic dodecadepsipeptide with a molecular weight of 1.2 kDa. The basic repeated amino acid sequence [D-*O*-Leu D-Ala L-*O*-Val D-Val]_3_ is extremely stable towards heat, acid or digestive enzymes and, thus, the toxin can hardly be removed or inactivated [10,11,12]. Usually, the emetic form of disease is self-limiting and symptoms disappear after 6–24 h. Nevertheless, some severe and fatal outbreaks mostly related to liver failure are reported [10,13,14,15,16,17,18,19,20,21,22,23]. Due to the ubiquitous nature of the pathogen and its production of highly resistant spores, *B. cereus* is frequently found in various kinds of food [24,25,26]. Historically, starchy foodstuffs such as rice or pasta are connected to food intoxications with emetic *B. cereus*, but more recently evidence is growing that emetic *B. cereus* are much more volatile than once thought. The comprehensive analysis of a total of 3654 food samples obtained from suspected food-borne illnesses with a preliminary report of vomiting, collected over a period of seven years, revealed that emetic *B. cereus* strains were detected in a broad diversity of foods, including vegetables, fruit products, sauces, soups, and salads as well as milk and meat products [7].

The second, diarrheal form of food poisoning is also associated with a variety of different foodstuffs [27]. This form of disease manifests mainly in diarrhea and abdominal cramps, similar to food poisoning by *Clostridium perfringens* type A [5]. Symptoms occur after approximately 8–16 h. This incubation time is typical for toxico-infections, in which the toxins are produced by viable bacteria inside the human intestine [5,28,29]. Unlike cereulide, enterotoxins pre-formed in foods most likely do not contribute to the disease, as they are considered sensitive towards heat, acids or proteases. Thus, vegetative *B. cereus* and, especially, spores must be consumed. The infective dose is estimated between 10^5^–10^8^ cfu/g [11,30] or 10^4^–10^9^ cfu/g [9,29] vegetative cells or spores. The course of disease is mainly mild and—after approximately 12–24 h—self-limiting. Fatal outbreaks are only very rarely reported [31]. A food infection with enteropathogenic *B. cereus* can be seen as a multifactorial process, as a number of individual steps have to be considered before the onset of the disease, including prevalence and survival of *B. cereus* in different foodstuffs, survival of the stomach passage, germination of spores, active movement towards and adhesion to the intestinal epithelium, enterotoxin production under intestinal conditions, as well as the influence of consumed foods and the intestinal microbiota on these processes. We recently summarized these steps in a separate review [27]. Nevertheless, production and action of the enterotoxins are of the upmost relevance for the course of the diarrheal disease. Three main, pore-forming protein enterotoxins are known, which are the tripartite hemolysin BL (Hbl) [32] and non-hemolytic enterotoxin (Nhe) [33], as well as the single protein cytotoxin K (CytK) [31]. Progress made in studies from the early 1990s until today on highly variable, strain-specific enterotoxin production (distribution, genetic organization, gene expression and toxin secretion), as well as on the mode of action and the effects on target cells of these pore-forming enterotoxins, is depicted in detail in the present review. The two three-component enterotoxin complexes Hbl and Nhe are the focus of attention. In addition, the latest findings regarding cereulide and CytK are also summarized, as well as known methods for toxin detection and prevention of illness, and the possible contribution of further secreted virulence factors to the diarrheal form of disease.

## 2. Distribution of Toxin Genes

### 2.1. Prevalence among Isolates from Environment, Foods and Outbreaks

*B. cereus* is a ubiquitous soil bacterium and can thus be found worldwide in the ground, in dust, or on different foods. Early studies pointed to an occurrence of diarrheal or emetic outbreaks according to country-specific dietary habits, with the emetic form manifesting in Great Britain or Japan, and the diarrheal form rather in Northern Europe or the USA [34,35]. Lately, both syndromes have been reported from all over the world. Basically, emetic strains are found less frequently in foods as well as in the environment than enteropathogenic strains [27,36,37]. In a multitude of studies, new isolates were screened for the presence of the toxin genes *nhe (ABC)*, *hbl (CDAB)*, *cytK (1,2)*, *entFM*, and *ces*. In some studies, the presence of *bceT* (enterotoxin T) was also assessed; however, its enterotoxic capacity is disproven [38,39,40]. Virulence/enterotoxin gene patterns are compiled for *B. cereus* which has been mainly isolated from foods, but also from clinical, soil and environmental samples worldwide. Generally, those patterns are highly diverse [41,42,43,44,45,46,47].

Common distribution of the toxin genes is approximately 85%–100*% nhe (ABC)*, approximately 40%–70% *hbl (CDA)*, approximately 40%–70% *cytK-2*, very few *ces*+, typically no *cytK-1*+, and—if tested—approximately 60%–100% *entFM*, which has been detected in studies from Europe [44,48,49,50,51,52,53,54], South America [55,56], North America [41,57], Asia [58,59,60,61,62,63,64,65] and Africa [66,67,68]. Nevertheless, in some studies, a connection was established between toxin gene patterns and geographical location of the isolates. Drewnowska et al. found that strains possessing *nheA*, *hblA* and *cytK-2* were predominant in regions with arid hot climate, and were comparably rare in continental cold climates [69]. This is supported by other studies suggesting that geographic origin might have an impact on the conservation of *hblA* among *B. cereus* populations [70,71,72]. Zhang et al. also claim a “regional feature for toxin gene distribution” [73].

Besides geographical location, toxin gene patterns seem to be also influenced by the kind of foodstuffs analyzed. For instance, Berthold-Pluta et al. found higher prevalence of *nhe*+ and *hbl*+, but lower prevalence of *ces*+ strains in food products of animal than of plant origin [74]. Rossi et al. showed that strains from dairy products had significantly lower *cytK-2* and *hblCDA* prevalence than strains from equipment or raw milk [75], and Hwang and Park found *hbl* in >95% of tested ready-to-eat (RTE) foods, but only in 30% of infant formulas. Furthermore, the prevalence of *cytK-2* was comparably low in the latter food [76].

Studies were also conducted comparing food related and food poisoning related strains. Santos et al. showed that food poisoning strains had a higher occurrence and higher genetic diversity of *plcR-papR*, *nheA*, *cytK-2*, *plcA*, and *gyrB* genes than strains isolated from soil or foods [77]. *CytK* and the combination *hbl-nhe-cytK* were more often found among food poisoning related than among food related strains [51,52,78].

Generally, all *B. cereus* isolates can be categorized into seven different toxin profiles: A (*nhe*+, *hbl*+, *cytK*+), B (*nhe*+, *cytK*+, *ces*+), C (*nhe*+, *hbl*+), D (*nhe*+, *cytK*+), E (*nhe*+, *ces*+), F (*nhe*+), and G (*cytK*+) [48]. In fact, the *hbl* genes alone or a combination of *ces* and *hbl* have only been reported for the very few emetic *Bacillus weihenstephanensis* isolates described so far [79]. There are further studies showing “unusual” results, particularly low or no prevalence of *nhe* [45,74,80,81,82,83,84] or extraordinarily high prevalence of *hbl* [76,85,86,87,88] or *ces* [89], which must be interpreted cautiously, especially as *nhe* is well known for its molecular heterogeneity [48,51,52]. Thus, the choice of detection methods, especially primer pairs for *nhe*, can have a crucial influence on the results.

However, it has to be mentioned that the presence of enterotoxin genes or a certain toxin gene profile does not necessarily allow conclusions on the toxic activity of a *B. cereus* isolate [53,90]. In our own studies, we chose pairs of strains with an identical toxin gene profile, but one strain exhibited high and the other low toxic activity both under routine laboratory and simulated intestinal growth conditions [91,92]. The reasons for this are so far not completely understood, but it is believed that highly variable and strain-specific mechanisms in toxin gene transcription, posttranscriptional and posttranslational modification and protein secretion are involved, which are summarized in Section 4.1.2.

### 2.2. Presence within the B. cereus Group

In many of the studies mentioned in Section 2.1, often only *B. cereus sensu lato (s. l.)* strains are investigated, meaning there is no differentiation between the members of the *B. cereus* group. In routine microbiological diagnostics, only “presumptive” *B. cereus* are detected on selective culture media according to international standards (ISO 7932:2005-03) [93,94]. The *B. cereus* group comprises at least eight species: *B. anthracis*, *B. cereus sensu stricto (s. s.)*, *B. thuringiensis*, *B. mycoides*, *B. pseudomycoides*, *B. weihenstephanensis*, *B. cytotoxicus* and *B. toyonensis* [95,96,97,98]. Additionally, more and more species such as *B. wiedmannii*, *B. bingmayongensis*, *B. gaemokensis*, *B. manliponensis*, and others are described [99,100,101,102,103]. Generally, they exhibit high genetic similarities and, thus, it has been suggested that they be considered as one species [5,104,105] or to completely change the taxonomic nomenclature of the *B. cereus* group [106]. Species definition is historically based on phenotypes or clinical and economical relevance. While the unique characteristics of *B. anthracis*, emetic *B. cereus* and *B. thuringiensis* are located on plasmids [105], the enterotoxins are chromosome-coded and can thus be present throughout the *B. cereus* group. This is particularly problematic for the assessment of *B. thuringiensis*, which is frequently used as biopesticide worldwide [107,108,109]. *B. thuringiensis* has been isolated from a variety of foodstuffs and the presence of the enterotoxin genes *nhe*, *hbl* and *cytK-2* has been shown, with similar percentages as for *B. cereus* [57,60,72,90,110,111,112,113,114,115,116,117,118,119,120,121,122,123,124,125], while *ces* genes have not been found [126,127]. Enterotoxin production and cytotoxic activity have also been shown [57,113,114,116,117,123,128,129,130,131], and *B. thuringiensis* could therefore be involved in food poisoning outbreaks [132]. Consequently, it was debated whether the *B. thuringiensis*-associated biopesticides represent a risk for public health. To clarify this question, there is a demand for simple methods enabling a clear discrimination between *B. cereus* and *B. thuringiensis* in routine food and clinical diagnostics as well as for unequivocal identification of the strains used as biopesticides [126].

Next to *B. cereus* and *B. thuringiensis*, further species of the *B. cereus* group were isolated from foods and the presence of enterotoxin genes was proven, such as *B. anthracis* [48], *B. mycoides* [42,43,48,70,71,133,134], *B. pseudomycoides* [42,71], *B. toyonensis* [135], and *B. weihenstephanensis* [42,48,71,136,137]. It has also been shown that *Bacillus* spp. outside the *B. cereus* group can harbor one or more enterotoxin genes [138,139]. For instance, Mäntynen and Lindström found *hblA*+ *B. pasteurii* DSM 33, *B. smithii* DSM 459, and *Bacillus* sp. DSM 466 [70]. *Nhe* and/or *hbl* genes were also detected in *B. amyloliquefaciens*, *B. circulans*, *B. lentimorbis*, and *B. pasteurii* [140]. On the other hand, From et al. found no enterotoxin genes outside the *B. cereus* group in the strains analyzed [141].

According to MLST (multi-locus sequence typing), AFLP (amplified fragment length polymorphism) and whole genome sequencing, the *B. cereus* group was first assigned to three phylogenetic groups (clades) [142], then seven (*panC* types) [96], and later nine [120], which do not correlate with species definition [105]. Prevalence of enterotoxin genes and their profiles were also compared to phylogenetic groups. *B. cereus* isolates from dairy products in Brazil with approximately 50% *cytK-2* and *hbl*, and approximately 85% *nhe* were mostly assigned to phylogenetic group III. Group IV and V showed significantly higher prevalence of *hblCDA* and group IV showed additionally higher prevalence of *cytK-2* [75,96]. In another study on dairy isolates, strains of clade IIIc had no *hblCDA* operon, while strains of clade IV carried it and produced the Hbl toxin, whereas strains of clade VI carried the gene but did not produce the toxin [120]. Furthermore, a broad distribution of enterotoxin genes among seven phylogenetic clades, in which dairy-associated isolates were divided, was shown [90]. Okutani et al. investigated the genomes of 44 *B. cereus* group isolates from soil, animal and food poisoning cases in Japan. Strains were assigned to four different *panC* types and five different clades. The *nhe* operon was found in all strains tested, while *ces* was detected only in the food poisoning strains. When the presence or absence of virulence-associated genes was statistically analyzed, the majority of soil and animal isolates was part of overlapping clusters, while three of the four food poisoning isolates formed a distinct cluster [143]. Furthermore, the *hbl* and the *ces* genes were significantly correlated with the phylogenetic group [143,144]. Several further studies suggested that the toxic potential of *B. cereus s. l.* strains depends rather on the phylogenetic group than on the species [96,120,145].

## 3. The Emetic Toxin Cereulide

In this section, key features of the highly bioactive depsipeptide toxin cereulide are presented, its biosynthesis and mode of action are briefly discussed, and an overview of diagnostic methods is given. For a more detailed overview from the perspective of the food industry, it is referred to a recent review of Rouzeau-Szynalski et al. [146] and in-depth insights into genetics and regulatory circuits directing cereulide biosynthesis are provided by Ehling-Schulz et al. [37].

### 3.1. Characteristics of Cereulide and Its Assembly via the Non-Ribosomal Peptide Synthetase Ces-NRPS

**Figure 1:**
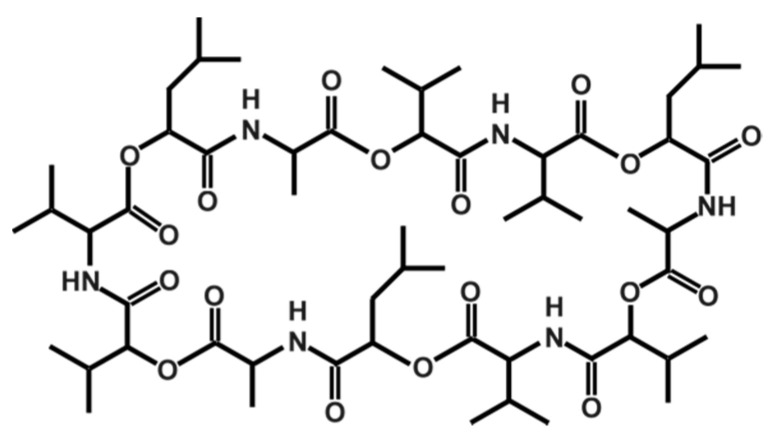
Structure of the depsipeptide toxin cereulide, the causative agent for the emetic type of *B. cereus* food-borne intoxications.

Cereulide, which was originally described by Agata et al. in 1994 [147], is a potassium binding depsipeptide that structurally resembles the ionophore valinomycin [148,149,150,151]. Due to its structure, which is characterized by alternating peptide and ester bounds (see Figure 1), it is highly lipophilic and extremely heat resistant and stable from pH 2 to 11. It does not lose activity even after two hours at 121 °C [12]. Because of its small size, it cannot be removed by normal hygienic procedures in food processing, such as bactofugation or filtration, nor will it be inactivated by heat treatments. Because of its resistance to cleavage by pepsin and trypsin, it will not be inactivated during stomach passage in the host [10]. Thus, it is of the utmost importance to prevent its synthesis in food production and processing. There are some indications that certain food additives, such as polyphosphates, have a specific inhibiting effect on cereulide synthesis while other food ingredients stimulate it [7,152] (see also Section 3.2).

More recently, several isoforms of cereulide have been described [153], which vary significantly in their cytotoxic potential. For instance, isocereulide A shows about 10-fold *in vitro* cytotoxicity compared to wild type cereulide, while isocereulide B does not show any cytotoxicity at all. These differences in cytotoxicity might be explained by different membrane activity of the isocereulides, as revealed by *in vitro* lipid bilayer studies [153]. The latter results foster the hypothesis that the ionophoric membrane activity of cereulide is one of the key features of its bioactivity (for more details see Section 3.3).

**Figure 2:**
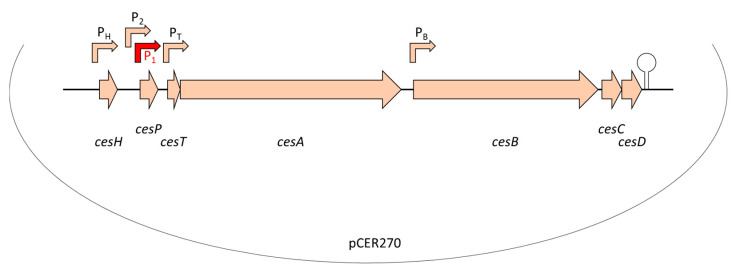
Genetic organization of the *ces* locus encoding the genetic determinants essential for non-ribosomal assembly of the cereulide toxin, located on the pX01-like mega-plasmid pCER270. For detailed description of the *ces* locus see text and [154,155,161,162]. Promoters are indicated by arrows. The main promoter P_1_ (indicated in red) drives the polycistronic transcription of the *ces* operon. A hairpin indicates the terminator. Abbreviation: *cesH*, a hydrolase/esterase; *cesP*, a phosphopantetheinyl transferase; *cesT*, a type II thioesterase; *cesA* and *cesB*, structural cereulide synthetase genes; *cesC* and *cesD*, ABC transporter.

Similar to valinomycin and other peptide antibiotics, cereulide is assembled by a non-ribosomal peptide synthetase (NRPS), called Ces-NRPS [148,154,155]. NRPSs are huge multienzyme intracellular machineries, which are often genetically organized into gene clusters [156]. Apart from the structural synthetase genes, the corresponding genetic loci often contain genes involved in the activation of the NRPS as well as putative ABC transporters, which are thought to be involved in peptide export and may confer self-resistance [157]. In addition, NRPS genetic loci may contain or be flanked by accessory genes encoding enzymes responsible for monomer modifications [158] or type II thioesterases ensuring correct peptide assembly [159]. The *ce*s genes are encoded on the pCER270 mega-plasmid, which shares it backbone with the pX01 toxin plasmid from *B. anthracis* [154,160]. The 24kb *ces* locus comprises a total of seven coding sequences (CDSs) [154] (see also Figure 2): *cesA* and *cesB*, the structural cereulide synthetase genes, which are flanked on the 5′ end by *cesH*, a putative hydrolase; *cesP*, a phosphopantetheinyl transferase (PPtase) involved in the activation of the cereulide synthetase; and *cesT*, a type II thioesterase thought to be involved in removal of mis-primed monomers. On the 3′ end of the *ces* locus two additional CDSs, designated *cesC* and *cesD,* are located that encode an ABC transporter. Very recently, it was shown that this ABC transporter is not only responsible for toxin export but also plays an essential and direct role in cereulide biosynthesis, by tethering the CesAB synthetase to the cell membrane [161]. *In vivo* studies revealed that CesAB colocalizes with CesCD on the cell membrane and BACTH (bacterial adenylate cyclase-based two-hybrid system) and mutation studies provided evidence that specific domains within the CesAB synthetase are interacting with CesC. Complementary *in vitro* as well as *in silico* studies suggested that this novel role of an ABC transporter, beyond the canonical function, discovered in emetic *B. cereus,* might represent a conserved mechanism involved in the biosynthesis of microbial natural products. This may facilitate the discovery of new bioactive metabolites [161].

### 3.2. Regulation of Cereulide Biosynthesis

Since production of secondary metabolites via NRPSs is metabolically very costly, these multi-enzyme machineries are usually tightly regulated [37]. Six out of the seven CDSs in the *ces* locus are transcribed as polycistron from the main promotor P1, located upstream of *cesP* (see Figure 2). This ensures an orchestrated transcription of the respective genes, which show a tightly regulated transcription peak in late exponential phase [163,164]. Additional promotors have been found in the *ces* locus, but their exact role in *ces* transcription and regulation is still elusive [162]. The putative hydrolase *cesH*, located in the 5′ region of the *ces* locus, is transcribed by its own promotor. Using a mutagenesis approach, Lücking et al. showed that CesH, which structurally belongs to the 6_AlphaBeta_hydrolase subgroup of the alpha/beta-hydrolase fold superfamily, inhibits cereulide synthesis [165]. CesH overexpression led to a cereulide-negative phenotype and transcriptional analysis of the CesH overexpression mutant resulted in strongly down-regulated *cesA* mRNA levels, indicating that CesH is involved in the timing of Ces-NRPS expression and cereulide assembly. Thus, it could be assumed that it functions as closing signal [165]. This hypothesis is fostered by the transcriptional kinetic of *cesH* showing highest *cesH* expression in stationary growth, whereas the other genes of the *ces* locus are transcribed in earlier growth phases [166]. However, since a direct action of a hydrolase as a transcriptional regulator is rather unlikely, it is tempting to speculate that CesH acts indirectly by degrading quorum sensing signaling molecules or metabolites, which may impact *ces* transcription in later growth phases. The findings are corroborated by a recent study. In line with the results from Lücking et al. [165], demonstrating that *cesH* inactivation leads to accelerated cereulide production, Tian et al. [167] found increased cereulide production in a ∆*cesH* mutant. Recombinant CesH was reported to possess esterase activity against para-nitrophenyl acetate but, so far, attempts to show esterase activity of CesH against cereulide have failed [167]. Thus, further studies will be necessary to decipher the exact role and mechanism of CesH in cereulide biosynthesis.

Previous work demonstrated that several key transcription factors of the chromosome play a pivotal role in the onset and steep increase of the transcription of the polycistronic *cesPTABCD* genes. Knockout as well as *in vitro* promotor binding studies [163,164] showed that the *ces* genes are not under control of PlcR, a pleiotropic regulator known to play a central role in enteropathogenic *B. cereus* [168,169] (see also Section 4.1.2). By contrast, the transcriptional regulator CodY, which acts as intracellular sensor of the energetic cell status and senses branched-chain amino acids [170], plays a key role in cereulide synthesis and tightly links toxin production to cell metabolism [37,163]. Furthermore, it has been shown that the transition state transcriptional regulator AbrB suppresses *ces* transcription in the early growth phase and thus couples cereulide synthesis to the developmental cell status and the Spo0A-AbrB regulatory circuit [37,164].

Although transcriptional regulators play a vital role in the control of cereulide biosynthesis, other realms of regulation must be taken into consideration. For instance, there is no strict co-linearity between *ces* transcription and actual cereulide toxin production [163,166]. Thus, posttranscriptional regulatory mechanisms must contribute to the complex network of directing and controlling cereulide biosynthesis. Generally, NRPSs are produced as apo-carrier proteins and 4′-phosphopanthetheinyl transferases (PPTases) must activate the NRPS modules by catalyzing the transfer of a coenzyme A-derived 4′-phosphopantetheine moiety to the peptide carrier protein (PCP) domains of the NRPS in order to convert them into their holo-form [171]. The *ces* gene locus encodes a PPTase, designated CesP [154], but unexpectedly a *cesP* knockout mutant was reported to still be able to produce cereulide [165], although functionality of CesP has been proven previously [148]. Only after disruption of an additional, chromosomally encoded PPTase (designated *ppt*) was a toxin-negative phenotype observed, indicating that this PPTase can function as a redundant CesP-PPTase in cereulide biosynthesis [165]. Thus, in cereulide biosynthesis there is not only chromosomal-plasmid crosstalk on a transcriptional level, but also on a posttranslational level, highlighting once more the complexity of the regulatory circuits governing cereulide production. The recently discovered novel and essential role of the ABC transporter CesCD in cereulide biosynthesis, which goes beyond its canonical export function, adds another level of complexity to the posttranslational regulatory circuits in cereulide formation [161]. Since cereulide is a highly bioactive ionophore, it could be hypothesized that it would be beneficial for the cells to orchestrate both biosynthesis and export of the toxin.

However, not only intrinsic but also extrinsic, environmental factors can have a significant impact on cereulide production capacity [172]. A comprehensive study showed that temperature is a cardinal parameter for cereulide production, which exerts control on posttranscriptional level [173]. Notably, maximum growth rate and maximum cereulide production were found to be decoupled, indicating that solely the number of bacteria or growth rates are no suitable parameters to predict the risk of cereulide production. Furthermore, the study of Kranzler et al. [173] revealed not only that the total amount of cereulide produced was highly temperature-dependent, but temperature also significantly impacted the formation of the recently identified cereulide isoforms. The production of isocereulide A, which is about 10-fold more cytotoxic than cereulide, was specifically supported at low temperatures (12 and 15 °C), whilst the production of the non-toxic isocereulide B was supported by ambient temperatures [173]. The latter must be taken into consideration in terms of food safety and predictive microbiology. Apart from temperature, other food industrial relevant extrinsic parameters, such as oxygen, pH or the food matrix, could also have a significant impact. However, further study will be necessary to fully understand the impact of specific extrinsic factors (and their interplay) on cereulide and isocereulide production in complex systems such as foods. Generally, there are indications that a neutral pH, high content of starch, carbohydrates, vitamins and trace elements support cereulide production, while low oxygen levels and high NaCl concentrations have a rather negative impact [7]. A detailed overview of the current knowledge of food industrial relevant external parameters is provided in a recent review by Rouzeau-Szynalski et al. [146]. There are also some indications that certain food additives, such as polyphosphates, could affect cereulide production in a specific manner by a hitherto unknown mechanism [152]. Furthermore, it has also been reported that specific antibiotics, which provoke the production of small colony variants (SCVs), could lead to dysregulation of cereulide synthesis resulting in derailed cereulide levels [174].

### 3.3. Mode of Action and within Host Translocation

Food-borne intoxications caused by cereulide are characterized by heavy episodes of vomiting shortly (15 min to 6 h) after consumption of contaminated foods, which are accompanied by nausea [10]. Based on results from a *Suncus murinus* animal model [6], it has been assumed that cereulide interacts with 5-HT3 serotonin receptors leading to the stimulation of the afferent vagus nerve and subsequent triggering of the vomiting center in the medulla oblongata. Usually symptoms decline after 24 h, but more severe intoxications are increasingly reported [7,14,18,23]. Due to its high ionophoric activity, cereulide can lead to rhabdomyolysis, liver damage and serious multiorgan failures. Low doses of cereulide have been reported to cause dysfunction in beta-cell lines [175] and to be detrimental towards isolated porcine pancreatic Langerhans islets [176]. Furthermore, by using whole mouse pancreatic islets and Min6 cells as model, it was shown that sub-emetic doses of cereulide have a direct impact on the insulin secretory machinery, suggesting that it may contribute to the ongoing diabetes endemic [175,177]. Recently it was reported that cereulide can co-occur with other microbial toxins, such as mycotoxins, in cereal-based foods [178]. Based on the results from *in vitro* co-cultivation studies of cereulide and the mycotoxin deoxynivalenol (DON), it was hypothesized that frequent consumption of cereal-based foods co-contaminated with cereulide and DON may cause synergistic cytotoxic effects and may alter the immune response in the human intestine [179]. However, since these results are only based on *in vitro* studies, further *in vivo* studies, using a suitable animal model, will be necessary to assess the actual risk associated with long-time dietary exposure to cereulide or the risks linked to co-exposure of cereulide and other microbial toxins.

As pigs are a well characterized model to study the physiological effects and toxico-kinetics of mycotoxins [180,181], which are structurally similar to cereulide, we explored their potential to gain first insights into uptake, resorption and translocation of the cereulide toxin within the body. To this end, piglets were orally challenged with different doses of cereulide [182]. The clinical symptoms mimicked those known from severe human intoxication cases, including lethargy, seizures, and convulsions [14,15,17,18,19], suggesting an involvement of the central nervous system (CNS). A part of the ingested cereulide toxin was absorbed and was distributed within the body, while the other part was rapidly excreted with the feces, indicating that screening of fecal samples by stable isotope dilution assay liquid chromatography mass spectrometry (SIDA LC-MS) may represent a suitable method for detection of cereulide intoxications (see Section 3.4). The results from the chronic trial indicated a bioaccumulation of cereulide in certain tissues and organs, such as kidney, liver, muscles and fat tissues [182]. Furthermore, it was demonstrated that cereulide can cross the blood-brain-barrier (BBB) and directly act on the brain [182], which may explain the cerebral effects reported from severe human intoxication cases. For instance, an 11 year old boy in Japan showed acute encephalopathy after consumption of cereulide-contaminated food [15]. The results from the pig intoxication model, as well as the clinical data from case reports, including the rapid onset of symptoms, indicate that the CNS is an important target for cereulide, which warrants further research to fully decipher the mode of action of the cereulide toxin.

### 3.4. Diagnostic Toolbox for Cereulide

**Figure 3:**
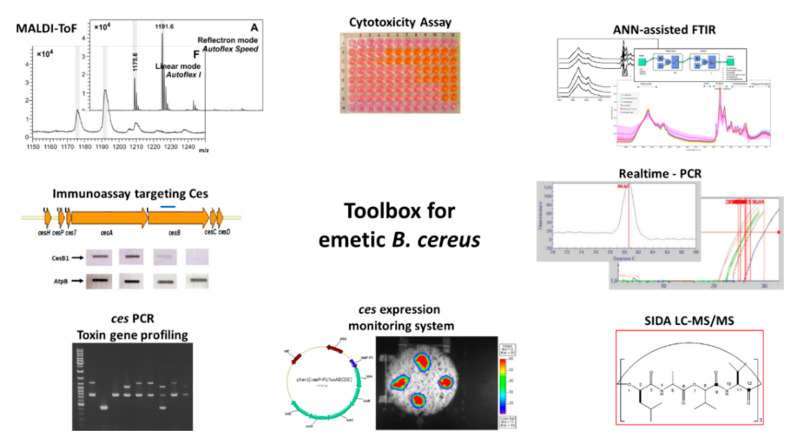
Toolbox for research and diagnostics of emetic *B. cereus* and the cereulide toxin. Several tools have been developed, which allow the identification of emetic *B. cereus* by means of molecular methods, such as conventional polymerase chain reaction (PCR) and real time PCR, mass spectrometry and spectroscopy, such as Matrix Assisted Laser Desorption Ionization-Time of Flight Mass Spectrometry (MALDI-ToF MS) and artificial neural network assisted Fourier transform infrared spectroscopy (ANN-assisted FTIR). Furthermore, tools have been developed to monitor the *ces-NRP*S (Non-Ribosomal Peptide Synthetase) expression on a transcriptional and translational level, such as a *lux*-promotor transcription assay and an immunoassay targeting the cereulide synthetase. In addition, mass spectrometry methods for quantitation of the cereulide toxin and isocereulides and cell culture-based assays for cytotoxicity studies are available, such as stable isotope dilution assay liquid chromatography mass spectrometry (SIDA LC-MS) and Hep-2 cell culture assays. Details of the respective methods are provided in Section 3.4. Images for the composite figure are based on the following publications: [7,36,48,162,165,183,184,185].

Throughout the last two decades, considerable progress has been made in the development of diagnostic tools for detection and identification of emetic *B. cereus* as well as for the identification and quantitation of the cereulide toxin and its derivatives. Furthermore, methods have been developed for transcriptional and translational analysis of the Ces-NRPS. An overview of tools available for cereulide diagnostics and research is provided in Figure 3.

The identification of the *ces-NRPS* gene cluster [155] was a breakthrough in cereulide research and diagnostics. Based on the genetic determinants responsible for non-ribosomal assembly of cereulide, PCR systems for the identification of emetic *B. cereus* and toxin gene profiling [48,186,187], as well as TaqMan probe-based real time PCR systems for diagnostics [185,188], have been developed. Furthermore, a *lux* promotor fusion bioluminescence reporter system for non-invasive real-time monitoring of cereulide synthetase promoter activity in different environments was designed [166]. The reporter system was successfully applied to classify food matrices (*n* = 70) into risk categories, ranging from low-risk to high-risk food [7], and to investigate the effect of food additives on cereulide production [152]. Apart from being a valuable tool in food microbiology, the *lux* reporter system is also a suitable tool for monitoring *ces* expression *in vivo* in the frame of mutagenesis studies [163,164]. The Ces-NRPS-specific antibody targeting the CesB1 module (see Figure 2), which became recently available [165], allows the investigation of the Ces-NRPS machinery on a translational level for gaining insights into posttranscriptional regulatory mechanisms involved in cereulide biosynthesis.

Apart from the progress made in molecular diagnostics of emetic *B. cereus*, the progress made in mass spectrometry and FTIR spectroscopy in recent years has also significantly improved differential diagnostics of emetic *B. cereus*. Recently, it was reported that emetic *B. cereus* and non-emetic *B. cereus* group strains can be differentiated by Matrix Assisted Laser Desorption Ionization-Time of Flight Mass Spectrometry (MALDI-ToF MS) based on certain biomarkers [189,190], and machine learning empowered FTIR spectroscopy was successfully employed to discriminate emetic *B. cereus* and other *B. cereus* group members using metabolic bacterial fingerprints [36]. In addition, MALDI-ToF MS was also found to be suitable for direct detection of cereulide in emetic *B. cereus* isolates [184]. For direct identification and accurate quantitation of cereulide in complex matrices such as foods [183,191] or clinical specimens [182], a SIDA LC-MS method was established [183], which provided the basis for an EN-ISO method (ISO 18465:2017) [192] for routine diagnostics. In addition, a SIDA-UPLC-MS/MS method for multiparametric quantitation of cereulide and isocereulides A-G in foods was established [193] to include the most prominent isocereulides [153] in cereulide diagnostics. Application of the latter assay in the investigation of two food-borne outbreaks linked to cereulide revealed that isocereulides were indeed present in considerable amounts in the contaminated food remnants, suggesting that isocereulides may play a yet to be explored role in the severity of food-borne intoxications.

The set of tools for research on emetic *B. cereus* is complemented by bioassays to investigate the *in vitro* cytotoxicity of the cereulide toxin and isocereulides using immortalized cell lines, such as Hep-2 or Hep-G2 cells, or, less frequently, CaCo-2 or HeLA cells [8,51,153,179,194,195]. Furthermore, boar sperms have been employed to demonstrate mitochondrial damage by cereulide [196,197].

## 4. The Diarrheal Enterotoxins

In this section, several features of the three main diarrheal enterotoxins Hbl, Nhe and CytK are discussed, such as their genetic organization, gene expression, toxin secretion, and mode of action. As Hbl and Nhe are both tripartite enterotoxin complexes sharing some similarities, but also significant differences, especially in their mode of action, they are summarized in Section 4.1. The β-barrel pore forming, single protein CytK is reviewed in Section 4.2.

### 4.1. The Tripartite Enterotoxins Hbl and Nhe

#### 4.1.1. Organization and Evolution of the Enterotoxin Genes

**Figure 4:**
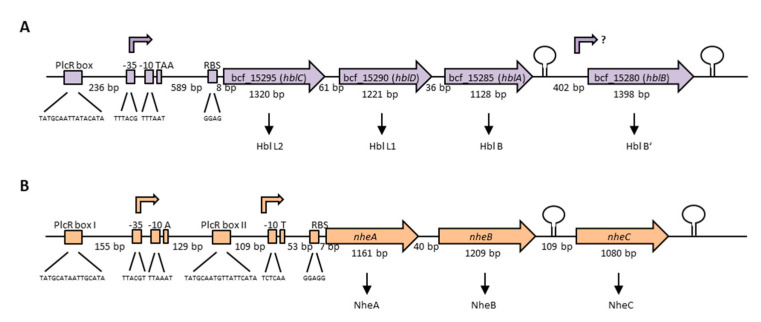
Genetic organization of the operons encoding the tripartite enterotoxins of *B. cereus*. (**A**) The *hblCDAB* operon of strain F837/76, encoding the proteins Hbl L2, L1, B and B’ [198,199]. Upstream and downstream of *hblB,* a stem loop was identified [204]. The promoter region, as well as the transcriptional start TAA is shown [201]. 236 bp upstream of the promoter, a PlcR-box is located [202]. (**B**) The *nhe* operon of strain NVH 0075-95, encoding NheA, B and C [91,210]. Two PlcR-boxes as well as two possible transcriptional start sites exist [201,209,210]. Upstream and downstream of *nheC,* a stem loop was identified [207,210].

The enterotoxins Hbl and Nhe consist of three protein components each, which are encoded in operons on the chromosome. The Hbl components L2, L1 and B are encoded by *hblC*, *hblD* and *hblA*, respectively [198]. Interestingly, two variants of the *hbl* operon exist among different *B. cereus* strains, with the more common one bearing a fourth gene, *hblB*, which encodes a protein described as hemolysin BL binding component precursor or Hbl B’. Figure 4A illustrates the *hblCDAB* operon of *B. cereus* strain F837/76, from which the toxin was originally purified [32,199,200]. Transcription starts 601 bp upstream of the *hblC* gene and the binding sequence for the global regulator PlcR is shown [201,202]. The genes *hblC, D*, *A* and *B* consist of 1320, 1221, 1128 and 1398 bp, respectively, with separating nucleotides of only 61 bp between *hblC* and *D*, and 36 bp between *hblD* and *A*. Between *hblA* and *B* is a space of 402 nucleotides. Sequence analyses showed 21%–70% similarity (41–82% identity) between the four Hbl components, suggesting that the *hbl* operon has originated by duplication from one ancestor gene [202]. The authors further suggested that, most recently, *hblB* has been generated by duplication of *hblA* and a *C*-terminal fusion with another ORF (open reading frame), as the *C*-terminal part of *hblB* is not similar to *hblA*. *hblB* was first identified sequencing the *hblA* gene of strain F837/76 [203]. Ryan et al. first showed that *hblC, D* and *A* are co-transcribed and assumed this also for *hblB* [198]. On the other hand, a stem loop has been identified upstream and downstream of *hblB*, which might act as transcriptional terminator [204]. For a long time, *hblB* was considered a pseudogene, as the *hblCDA* transcript seemed to terminate within [5,201,205] or upstream [204] of *hblB*. Only in 2010 did secretome analyses under different redox conditions reveal that *hblB* is indeed transcribed, translated and exported at detectable levels in the early secretome of the *B. cereus* type strain ATCC 14579 [206]. Moreover, mRNA analyses pointed to the fact that *hblB* may be expressed as a single, monocistronic transcript with an own promoter structure and transcriptional start side.

The three Nhe components A, B and C are encoded by *nheA*, *nheB* and *nheC*, respectively [207]. Figure 4B shows the *nhe* operon of *B. cereus* strain NVH 0075-95, from which the toxin components A and B were originally purified after a large food poisoning outbreak in Norway in 1995 [33,208]. Only after sequencing of the NheA and B encoding operon was the existence of NheC proven [207]. The genes *nheA, nheB* and *nheC* consist of 1161, 1209 and 1080 bp, respectively. Between *nheA* and *nheB* is a space of only 40 bp, and between *nheB* and *nheC* 109 bp. Northern blotting revealed that all three genes encoding the Nhe toxin components are transcribed in one polycistronic mRNA [209]. Nevertheless, inverted repeats between *nheB* and *nheC* and after the *nheC* stop codon were identified by sequencing [207], and are shown as stem loops in Figure 4B. Furthermore, the intergenic region between *nheA* and *nheB* could be amplified, but any attempt to amplify the intergenic region between *nheB* and *nheC* did not succeed [209]. Interestingly, two promoter regions with two PlcR binding sites were identified upstream of *nheA* [54,91,201,209]. Additionally, unusually long 5′ untranslated regions exist upstream of the *hbl* and *nhe* transcription start sites, which were not found upstream of *cytK-1* or *cytK-2* [210]. Though the tripartite enterotoxins are known to be encoded on the chromosome, one study provided evidence for a second operon encoding all three Nhe components (42%–56% sequence identity) on a large plasmid in *B. weihenstephanensis* strain KBAB4 [211]. Evidence has been found that redundant genes in the *B. cereus* group are often carried on plasmids, but that their transcription is differently regulated [212].

In any case, total genome sequencing of selected enteropathogenic and apathogenic strains revealed the presence of two *nhe* operons in moderately toxic strain 14294-3 (M6) and low toxic MHI 226, isolated from ice cream and a milk product, respectively. Moreover, two *hbl* operons were detected in 14294-3 (M6), moderately toxic strain 6/27/S isolated from human feces, high toxic strain RIVM BC 126 from human feces, and low toxic strain RIVM BC 934 isolated from salad [54,91]. Duplication of the enterotoxin operons seems thus not to correlate with toxic activity. It has been shown in earlier studies that different strains can harbor both variants of the *hbl* operon (see above), and thus, express homologous sets of Hbl proteins. These are the more common Hbl L2, L1, B (and B’) from the *hblCDAB* operon and the rarer variants Hbl L2a, L1a and Ba from the *hblCDA* operon, each set of proteins with distinct properties. While the *hblCDAB* operon is highly conserved among different strains (sequence identities of 97%–99%), the *hblCDA* operon shows 75%–82% identity towards *hblCDAB* [91,211,213,214]. Our own studies also suggested high conservation of the *nhe* operon among different *B. cereus* strains (93%–99% similarity); however, these were all enteropathogenic, *ces*-strains from phylogenetic clades I and II [91]. An earlier study showed a novel Nhe variant in three *B. cytotoxicus* strains with only 80% protein sequence identity compared to known Nhe proteins [215]. In a study where 81 *B. cereus s. l.* strains were compared, it was shown that the upstream regions of *nheABC* were more conserved than the downstream regions [216]. Moreover, NheA protein sequences were sorted into four groups, according to their identities ranging from 100% to 78%. NheB protein sequences were most conserved, ranging from 100% to 87% identity in three groups. The least conserved component was NheC with 100% to 73% identity assigned to three groups of strains. As observed before, *B. cytotoxicus* strain NVH391-98 showed least conservation [216]. Earlier observations suggested that variations appear especially in sequence and length of the intergenic regions between *nheB* and *nheC* [5].

Sequence identities have not only been found between the three components of each enterotoxin, but also between the two toxins, with Hbl L2 and NheA sharing 23% sequence identity, Hbl L1 and NheB 40%, and Hbl B and NheC 25% [217]. Thus, it is speculated that the *nhe* and *hbl* genes arose from duplication of a single ancestor gene and that the latest duplication was the generation of *nheB* and *nheC* [5]. Böhm et al. investigated the evolution of chromosomally encoded enterotoxins. Phylogenetic analyses of 142 sequenced *B. cereus s. l.* strains revealed evidence for horizontal gene transfer, duplication, and frequent deletion of *hbl.* The duplicated *hbl* variant *hbla* was found in 22% of all genomes. Earlier studies found that the *hblCDAB* operon is part of an approximately 17.7-kb, 11 gene fragment, which has probably been acquired by transposal insertion [202,218]. For this, further evidence could not be found [54]. In contrast to *hbl*, duplication or deletion of *nhe* was hardly observed (0.02%). Stable horizontal transfer of the operon also seems to be rare; it is almost exclusively transmitted vertically [54]. Due to its altered structure, some studies speculated about an additional function of Nhe requiring interaction with further, so far unknown proteins [219], which is supported by the strict vertical evolution, the missing horizontal gene transfer, and the rare and instable duplication of the *nhe* genes. Loss or horizontal transfer of *nhe* has even been associated with a negative impact on the survival of the bacteria. Furthermore, horizontal transfer of *cytK* and *plcR* was observed, as well as frequent deletion of *cytK*. Altogether, the authors concluded that the evolution of the enterotoxins inside the *B. cereus* group is extremely variable, for so far unknown reasons [54]. Widespread lateral gene transfer within the *B. cereus* group and the great importance of horizontal gene transfer in the evolution of the enterotoxins have been suggested earlier, when 47 food-borne isolates were compared using MLST [220]. The authors also found a broad distribution of *nhe* and *hbl*, while *cytK* was limited to some phylogenetic clusters, and probably lost in distinct lineages. It was further concluded that gene loss is not linked to a particular species, and that the *hbl*, *nhe*, *cytK* (and *entFM*) genes were already present in a common ancestor of lineage I. Moreover, three toxin gene patterns showed a widespread coinheritance: *hbl* and *nhe*; *hbl*, *nhe* and *entFM*; and *nhe* and *entFM* [220]. Earlier, several studies already showed that horizontal gene transfer within B. *cereus s. l.* is generally possible, involving plasmids or further transposable elements [218,221,222,223,224]. Evidence has been found that massive gene exchange between plasmids and the chromosome, not restricted to distinct regions, occurred during evolution of the *B. cereus* group [212]. However, no *ces* genes have been found in the chromosome so far, and plasmid-mediated horizontal gene transfer of the emetic toxin, anthrax and insecticidal toxins is already known [54]. Rasigade et al. investigated 10 complete *B. cereus s. l.* genomes in search of genes under positive evolutionary selection and concluded that “adaptation to animal hosts, whether as pathogens, saprophytes or symbionts, is the major driving force in the evolution of the *B. cereus* group” [225].

#### 4.1.2. Enterotoxin Gene Expression and Toxin Secretion

Among the multitude of studies determining and identifying enterotoxin genes and pattern/profiles, it was repeatedly attempted to link them to cytotoxicity. By now, it is commonly accepted that the presence of enterotoxin genes hardly reveals evidence on the toxic potential of a certain isolate, especially as there are strains with identical enterotoxin gene profiles and even nearly identical toxin gene sequences producing highly variable amounts of toxins [91,226,227]. If not the enterotoxin genes, what lies beneath this extreme variability of toxin production? The answer is hidden in the complex, interwoven and not yet fully understood processes of enterotoxin gene transcription, posttranscriptional and posttranslational modification, and protein secretion.

The expression of the enterotoxin genes is highly complex and probably strain-specifically affected by environmental factors such as temperature, pH, oxygen tension, and nutrient (carbohydrate, nitrogen) availability, as well as by intrinsic factors such as the growth or the general energetic status of the cell [163,169,228,229,230,231,232,233,234]. Next to the PlcR-boxes (see Figure 4), binding sites for further transcriptional regulatory proteins such as CodY, ResD, Fnr, CcpA and SinR have been identified in the promoter regions of the *hbl* and *nhe* operons, which presumably control enterotoxin expression in a concerted action [169,210,230,233,235,236]. Many of the virulence factors secreted by *B. cereus* including Hbl and Nhe are under the control of the 34 kDa pleiotropic transcriptional regulator PlcR, which activates their gene transcription at the onset of the stationary growth phase [169,201,202,237,238]. Nutrient depletion leads to repression of *plcR* transcription itself via the sporulation regulator Spo0A-P [239]. Furthermore, the expression of *plcR* is positively autoregulated, but is also influenced by the YvfTU two-component system, whose encoding genes lie in proximity to *plcR* [238,240]. PlcR is part of a quorum-sensing system requiring also the 48 amino acid signaling peptide PapR, which is encoded 70 bp downstream of *plcR* and exported, processed and re-imported as heptapeptide PapR_7_ into the cell via the oligopeptide permease OppABCDF [168,241,242,243]. Evidence has been found that the secreted neutral protease B (NprB) is required for extracellular PapR processing [244]. At high cell densities, PapR is accumulated inside the cell and functions as autoinducer for PlcR. Especially the *C*-terminal regions of the two partners seem to be important for their interaction [244]. The PlcR-PapR complex can then bind the PlcR box, a specific target site in the promoter region with a conserved, palindromic sequence (TATGNANNNNTNCATA), which activates gene transcription [169,201,239,242]. Crystallization revealed an asymmetric, dimeric structure of PlcR complexed with PapR, including an *N*-terminal helix-turn-helix DNA binding domain and a *C*-terminal 11 helix domain interacting with the autoinducer. It was also suggested that this interaction causes further oligomerization of the PlcR dimers establishing a right-handed spiral interacting with the DNA [168]. Crystal structures of the apo-form of PlcR and the ternary complex with PapR and the PlcR box showed major conformational changes followed by binding of the helix-turn-helix domains in the major groove of the two half sites of the PlcR-box, revealing the mechanism of PapR-PlcR transcriptional activation [245]. Nonetheless, the PlcR-PapR system is also subject to strain-specific variations. Slamti et al. found different types of mutations in the *plcR* gene responsible for hemolysis- and lecithinase-negative phenotypes [246]. In *B. anthracis*, the PlcR regulon is silenced due to a nonsense mutation in *plcR*, which can be restored by the expression of a functional PlcR-PapR fusion protein [247,248]. PlcR activation due to PapR has also been shown to be strain-specific, involving especially the first amino acids of the peptide. Four classes of PlcR-PapR pairs were distinguished leading to four distinct pherotypes (based on pheromone-receptor association) among the *B. cereus* group [249]. Variability in enterotoxin transcription is further induced by the presence of two transcriptional start sites with two PlcR boxes upstream of the *nhe* operon [201,209]. Interestingly, newer studies have found a second quorum-sensing-system, consisting of PlcR-paralog PlcRa and the signaling peptide PapRa_7_, which is involved in oxidative stress response and cysteine metabolism. PapR variants were also able to activate PlcRa, which showed a connection between the PlcR and PlcRa regulons for the first time [250,251]. Recent studies focus on the inactivation of the PlcR-PapR quorum sensing system and, thus, the inhibition of virulence factor production due to synthetic PapR_7_ derivatives. Furthermore, it was found that especially proline and glutamic acid residues of PapR play an important role in PlcR regulon activation [252,253].

Next to PlcR, the pleiotropic regulator CodY is strongly involved in enterotoxin gene expression in *B. cereus* [163,254]. Generally, this conserved transcriptional repressor senses nutrient availability and the energy status of the cell in low GC Gram-positive bacteria by binding GTP or branched-chain amino acids. Under nutrient starvation, genes of the CodY regulon involved in motility, chemotaxis, catabolism, and virulence are derepressed in complex ways, directly or indirectly [170,255,256,257,258,259,260,261,262,263,264]. First studies with *codY* deletion mutants in *B. cereus* pointed to strong effects of the regulator on the PlcR-PapR quorum sensing system and assigned CodY a key role in pathogenicity, toxin production and adaptation to nutrient starvation [163,254]. On the one hand, CodY influences enterotoxin gene expression directly via binding to specific target sites in their promoter regions. Upstream of the *nhe* and *hbl* operons, long 5′ untranslated regions were detected, in which one (*hbl*) and even two (*nhe*) CodY binding site were identified [210]. Furthermore, the length of these regions also influences promoter activities. While PlcR binding sites are conserved inside the *B. cereus* group, higher variability has been found among the CodY recognition sites. This leads to diverging affinity of the regulator to its target promoter regions and thus can be one explanation for the high strain-specific variability in enterotoxin production [210]. On the other hand, CodY exerts influence on PlcR by regulating the re-import of the signaling peptide PapR. It has been shown that CodY controls the production of the oligopeptide permease OppABCDF as well as further Opp-like proteins [265,266].

In the promoter region of the *hbl* operon, three putative ResD and two putative Fnr binding sites are located, while two putative ResD and one putative Fnr recognition sites have been identified upstream of the *nhe* genes [210,229]. The two-component system ResDE was shown to control fermentative growth and enterotoxin expression under low oxidoreduction potential anaerobic conditions [229]. ResD is the response regulator and ResE the corresponding sensor kinase. ResD directly binds to the promoter regions of *hbl* and *nhe*, as well as *resDE*, *fnr*, and *plcR* [235]. It has also been shown that phosphorylation increases ResD interaction with *resDE* and *fnr* promoters, and that ResD and Fnr bind their target DNA in a concerted action. It was further suggested that ResD acts as anti-activator, and ResD-P as an activator of Fnr [235]. Fnr is a redox regulator necessary for fermentative growth under low oxidoreduction potential, as shown in an *fnr* deletion strain of *B. cereus*. Furthermore, expression of the *hbl* and *nhe* operons was significantly decreased in the mutant strain [234]. Interestingly, Hbl production was more strongly affected by the deletion of *fnr* than Nhe production [267]. It has been shown that monomeric apoFnr binds to the promoter regions of *hbl* and *nhe*, as well as *fnr*, *resDE*, and *plcR,* and thus, activates their expression [236]. Furthermore, Fnr is not only important for carbon source regulation in *B. cereus*, but also for the regulation of enterotoxin gene expression in response to carbohydrates [267]. It has also been proven that Fnr forms a ternary complex with ResD and PlcR. An Fnr monomer binds one [4Fe-4S]2+ cluster, which is, though, not required for DNA binding or formation of the complex [268]. Another intriguing fact is that Fnr positively regulates *Escherichia coli* ClyA (see Section 4.1.3) [269]. It has been concluded that *B. cereus* might induce enterotoxin gene expression as part of a compensatory metabolic pathway aiming to maintain the intracellular redox state in proximity to the intestinal epithelium [270].

The *hbl* and *nhe* operons are also controlled by CcpA-mediated catabolite repression. CcpA is a transcriptional repressor binding to promoter regions at specific, palindromic catabolite responsive elements (cre-sites), thus controlling glucose catabolism while repressing gluconeogenesis and alternative metabolic pathways [271]. Potential cre sites have been found upstream of the *hbl* and *nhe* operons [5,210,233]. During anaerobiosis, in the stationary growth phase and under glucose surplus, the expression of the operons is repressed [228,233]. Reversely, using sucrose or fructose resulted in enhanced Hbl and Nhe production compared to glucose as carbon source [272,273]. Similar to Fnr, CcpA seems to affect *hbl* and *nhe* expression differently, as deletion of *ccpA* resulted in higher expression of *nhe* compared to *hbl* [233].

Additionally, the phase-transition regulator SinR is involved in enterotoxin gene expression. SinR and its antagonists SinI, SlrA, and SlrR interactively regulate biofilm formation in *Bacillus subtilis* [274,275]. Deletion mutants revealed that Spo0A, AbrB and SinI/SinR control biofilm formation and swimming motility in *B. thuringiensis* [230]. Furthermore, *hbl* transcription was shown to be controlled by SinI/SinR together with PlcR. Interestingly, only a small subpopulation of cells in biofilms expressed *hbl*, which corresponded to *sinI* expression [230]. Furthermore, two SinR recognition sites were found upstream of the *hbl* operon, while one SinR binding site was identified upstream of the *nhe* operon, in close proximity to an Fnr binding site as well as a cre element [210]. An essential role of the *spo0A-sinI-sinR* regulatory circuit has also been shown for biofilm formation, cell differentiation, nematocidal activity, and bacteria-host interactions of *B. cereus* AR156 [276].

Beyond the six verified transcriptional regulators PlcR, CodY, ResD, Fnr, CcpA and SinR, further regulatory mechanisms of enterotoxin expression have been suggested. On the one hand, expression of *hblB* might be independent from the *hblCDA* operon due to its putative own promoter [204,206]. Microarray and qRT-PCR analyses already revealed two-fold upregulation of *hblB* in hyperflagellated swarm cells [277]. Furthermore, expression of *hblB* was found to be 126-fold down-regulated in the presence of mucin, while *nheB* and *hblA* were 100-fold and 110-fold up-regulated, respectively [278]. This observation confirmed a separate, so far unknown regulatory mechanism. Moreover, Clair et al. showed that Hbl and Nhe secretion is influenced by the OhrRA system, which is involved in response to redox changes. It was suggested that OhrA affects toxin secretion mainly post-transcriptionally and that OhrR might even be a transcriptional repressor for *hblCDAB* and *nheAB* [279]. A deletion mutant of transcriptional regulator RpoN (sigma 54) was impaired in NheA production [280]. Especially the unusually long 5′ non translated regions of the *hbl* and *nhe* operons might harbor additional regulatory mechanisms. Intergenic regions encoding small regulatory peptides or proteins are increasingly found in prokaryotes [281]. Upstream of the *hbl* operon, a potential ORF encoding a so far unknown protein has been identified [210]. Furthermore, these unusually long sequences might host temperature-sensitive RNA thermometers or metabolite-responsive riboswitches [210] which, however, have not been identified upstream of the enterotoxin genes in *B. cereus* so far.

It has also been shown that several host factors influence *B. cereus* enterotoxin expression. Besides temperature, oxygen tension, nutrient availability, growth phase etc. (see above), the host epithelium is of particular interest. Very recently, we identified massive transcriptional changes in *B. cereus* after contact with mucin, including genes encoding enterotoxins (Hbl, Nhe) and further putative virulence factors. The number of secreted enterotoxins was also increased in various *B. cereus* strains, depending on mucin concentration [278]. Previously, we showed that the secretome of CaCo-2 cells influences entero-toxin gene expression and toxin production [92]. Growing for only two hours in cell culture medium pre-incubated with CaCo-2 cells mimicking intestinal conditions, enterotoxin gene expression of different *B. cereus* strains was activated and total protein secretion was enhanced. Similarly, germination of spores from eight out of 11 enterotoxic *B. cereus* strains was triggered by differentiated CaCo-2 cells in an earlier study. The germinant, which still needs to be identified, is secreted by the cells, stable towards heat and proteolysis, and most likely bound or degraded by the spores [282]. Our own studies proved that the factor triggering enterotoxin production under simulated intestinal conditions also originates from the CaCo-2 cells, is used up by the bacteria, and affects high as well as low toxic *B. cereus* strains [92]. Furthermore, the molecule is small, <3 kDa, heat stable, is secreted by the cells long before contact with the bacteria, is not restricted to CaCo-2 cells, but is rather secreted by various cell lines from different organisms and compartments, independently from cell differentiation (unpublished data).

As mentioned above, *B. cereus* increases total protein and thus enterotoxin secretion when sensing the host environment [92,278]. The amino acid sequences of all Nhe and Hbl components contain *N*-terminal signal peptides for secretion, indicating toxin secretion via the Sec translocation pathway. Modification of this signal sequence in Hbl B led to loss of secretion and thus, to intracellular accumulation of the protein. It was additionally shown that sodium azide, an inhibitor of SecA, reduced secretion of the enterotoxins and enhanced their intracellular accumulation [283]. The Sec translocation machinery consists of SecYEG (pore), SecDF and the ATPase SecA. The number of enterotoxins in the secretome was clearly reduced in a Δ*secDF* mutant. Further enzymes such as chaperones or peptidases are also involved in the secretion process [284,285]. On the other hand, evidence has been found for an involvement of flagella in the export of several virulence factors. The flagellar export apparatus, which resembles the type III secretion system in Gram-negative bacteria, can also be used for secretion of non-flagellar proteins [286]. Motility and virulence are connected in *B. cereus* via flagella, as flagellin expression and motility were decreased in a *plcR* mutant [237,287]. Deletion of *flhA* and lack of flagella led to defective secretion and to intracellular degradation of Hbl, respectively [288,289,290]. Furthermore, *hblC* transcription was impaired in the *flhA* mutant [291]. The correlation between swarming and hemolysin BL secretion in 42 *B. cereus* group isolates confirmed this connection [289]. Similarly, an *flhF* (encoding a signal recognition particle (SRP)-like GTPase) deletion resulted in decreased secretion of flagellin, Hbl and further virulence factors, as well as in an increase of NheB and other virulence factors in the secretome [290,292]. This was confirmed by the differential expression of 118 genes during swarming, including flagellar genes and the *hbl* operon [277]. Very recently, it was shown that FlhF does not interfere with the expression of *hblC*, but that it binds the Hbl L2 protein and recruits it to the plasma membrane for secretion [293]. Thus, motility and flagella are intensely related to pathogenicity, and especially to Hbl secretion [290,292,293,294,295,296]. As the sizes of the Hbl components found in the secretome match those calculated for the proteins lacking the *N*-terminal signal peptides for secretion, it has been suggested that signal peptidases collaborate with the flagellar export system for Hbl secretion [296]. Secretion of Nhe seems to be rather independent from the flagellar export system and, thus, is mediated exclusively by the Sec translocation pathway.

Multiple attempts have been made to predict the toxic potential of newly isolated *B. cereus* strains [27,297]. This is a difficult venture, as factors such as toxin gene expression, secretion and protein stability are highly diverse among members of the PlcR regulon within one single strain [298]. Additionally, the different regulatory mechanisms from enterotoxin gene transcription to toxin secretion summarized in this section probably underlie extreme strain-specific variations.

#### 4.1.3. Structure and Mode of Action of the Enterotoxin Complexes

When hemolysin BL was originally purified from *B. cereus* strain F837/76 isolated from a postoperative wound [299], a molecular weight of 45, 36, and 35 kDa was postulated for the three components L2, L1 and B, respectively. First, only a binding and a lytic component were identified, which was later corrected to three components [204,300,301]. Hbl owes its name to its ability to lyse sheep erythrocytes [32,200]. As two Hbl encoding operons exist (see Section 4.1.1), distinct homologues of each protein varying in size can occur between *B. cereus* isolates or in one single isolate [54,213,214]. In early studies, it was shown that Hbl is involved in fluid accumulation in rabbit ileal loops, is active in vascular permeability tests, and has dermonecrotic, hemolytic and cytotoxic properties [32,200,213,302,303,304,305,306]. Interestingly, functional differences were found between Hbl and Hbla (see Section 4.1.1), as Hbla did not lead to the Hbl-typical ring-shaped hemolysis zone phenomenon on blood agar [213]. Though Hbl was the earlier enterotoxin to be discovered in *B. cereus*, determination of its toxic activity and mode of action has been difficult for a long time, as nearly all natural *B. cereus* isolates additionally bear and secrete Nhe (see Section 2.1). Furthermore, it was largely believed that Nhe or at least NheA might have an essential function for *B. cereus* [5,54], and several attempts to generate complete *nhe* deletion strains failed [217,307]. Further difficulties were faced creating recombinant Hbl components, and it was even suggested that they might be toxic for *E. coli* [198,203,304]. These issues were resolved years later, when functional recombinant Hbl proteins could be overexpressed in *B. anthracis* [204] and *E. coli* [308], and the *nhe* operon was successfully deleted in strain F837/76 [308].

NheA and B were first isolated from *hbl*-negative strain NVH 0075-95, which caused a large food poisoning outbreak in Norway [33,208]. Initially, NheC could not be identified, possibly due to its low expression compared to NheA and B, which has been proved in later studies. Basically, sequencing of the *nhe* operon led to the identification of *nheC,* and the corresponding protein was shown to be part of the Nhe toxin [207,209]. The molecular weight of the three components was determined as 41 kDa (NheA), 39.8 kDa (NheB) and 36.5 kDa (NheC) [207]. However, also for Nhe strain-specific variations exist [5]. All three recombinant Nhe protein components were successfully overexpressed in *B. anthracis* [204] and, just recently, in a eukaryotic cell-free protein expression system [309]. The Nhe and Hbl proteins share sequence identities, both between the three components of each complex and between the two enterotoxin complexes. Hbl L2 and NheA share 23% sequence identity, Hbl L1 and NheB 40%, and Hbl B and NheC 25%. Highest identity was found between NheB and NheC (44%) [217]. Similarities were also found when predicted transmembrane helices were compared. Of these, NheA and Hbl L2 have none, NheB and Hbl L1 have two, and NheC and Hbl B have one [5,207,310]. When all six enterotoxin components were recombinantly produced in *B. anthracis*, it was proven that they are not arbitrarily interchangeable among each other. Thus, the Hbl pore cannot be complemented by any Nhe component and vice versa [204].

As Hbl and Nhe showed no sequence homologies to any other known toxins, they could not be assigned to a group of toxins for some time. However, the crystal structure of Hbl B, which was determined in 2008, revealed great structural similarities to Cytolysin A (ClyA) from Gram-negative bacteria [311]. Thus, Hbl and Nhe were assigned to the ClyA superfamily of α-helical pore forming toxins, which was confirmed later by the crystal structure of NheA [217,219,311,312]. The 34 kDa, hemolytic and pore-forming protein ClyA or SheA (silent hemolysin A) or HlyE (hemolysin E) is one of the best characterized α-pore-forming toxins [313]. Gene expression of *clyA* is repressed by the nucleoid-associated protein H-NS, and positively influenced by the regulator SlyA, as well as under anaerobic conditions by the above mentioned Fnr [314]. Cytotoxicity evolves from the formation of 3 nm cation selective pores after cholesterol binding on the cell surface [315,316]. The monomer consists of a tail domain composed of four 80–90 Å α-helices (α-A-C and α-F) and the fifth, 35 Å α-G helix, as well as a head domain composed of two antiparallel β-sheets, the characteristic β-tongue [317]. In contact with lipids, cell membranes or detergents, the protein oligomerizes and forms ring-shaped structures acting as transmembrane pores [317,318]. The overall, 400 kDa pore complex consisting of 12 monomers is formed via huge conformational changes, reorganization of the hydrophobic core and alterations of the β-sheets and the loops in the α-helices [313,317,318,319]. The mechanism of pore formation is significantly different from other known toxins. First, linear oligomers of different sizes are built, then they assemble as pairs, which are able to directly close the pore complex ring, which makes pore formation extremely fast and efficient [313,319,320]. A current study shows that ClyA can permeabilize membranes even before complete pore formation, letting particles smaller than 400 Da pass through [321]. The full pore resembles a hollow cylinder of 130 Å height, an outer diameter of 105 Å and the smallest inner diameter of 35 Å. The inside is negatively charged underlying the cation selectivity of the pore [313,315]. The hydrophobic β-tongue is assumed to be inserted into the membrane first [319].

The crystal structure of Hbl B shows some remarkable similarities to ClyA, such as the large bundle of five α-helices and the head domain with hydrophobic β-sheets. Its size is approximately 90 Å × 40 Å × 30 Å [311]. Based on these similarities, the authors suggested that Hbl B alone might be able to oligomerize to a heptamer or octamer and form a pore. In that case, Hbl L1 and L2 were either responsible for conformational changes of Hbl B or for the stabilization of the head domain. It was also speculated that these toxin components might infiltrate the cell, similarly to anthrax toxin [311]. As mentioned, Hbl L2 itself does not possess hydrophobic sequences in its head domain. On the other hand, those of Hbl L1 are even longer than the hydrophobic regions of Hbl B. Thus, the theory came up that Hbl L1 might be able to form two complete transmembrane helices [110,219]. Due to the sequence identity of NheB and NheC towards Hbl B, homology models based on the Hbl B crystal structure were established. Their structures also contain a bundle of four α-helices wrapping around each other in left-handed supercoils, a shorter fifth carboxy-terminal helix, as well as a β-hairpin correlating with the hydrophobic regions of the proteins [217]. Resolving the crystal structure of NheA showed a shortened *N*-terminal α-helix compared to ClyA, which might point to a significant difference in pore formation. Despite a generally similar structure to Hbl B or ClyA, the head domain of NheA is significantly enlarged [219]. Furthermore, the typical hydrophobic β-hairpin is exchanged for an amphipathic hairpin connected to the main protein in a manner known from *S. aureus* β-pore forming toxins. Thus, the authors concluded that this β-tongue is not able to form a ClyA-like hydrophobic transmembrane helix, leading to the thesis that NheA, after binding to the NheB-C pro-pore, forms a transmembrane β-pore, analogously to *S. aureus* α-hemolysin [219,322]. Despite all homologies, this would be a fundamental difference in the mechanism of pore formation of Hbl and Nhe.

Hbl pore formation was first shown by osmotic protection assays [300]. The authors also suggested that all three Hbl components can bind individually to erythrocytes and form a “membrane attack complex”, which finally leads to cell lysis. Early experiments with rabbit ileal loops also suggested equimolar amounts of Hbl L2, L1 and B for toxic activity [304], or lower amounts of Hbl L2 or L1 compared to B for maximal hemolytic activity [305]. In these early studies, the discontinuous ring-shaped hemolysis zone phenomenon was also discovered, which is characteristic for Hbl [32,300,304]. When Hbl diffuses through blood agar from a particular point, hemolysis is observed at a certain distance from that point. This is presumably caused by the variable diffusion velocity of the three single Hbl components leading to a continuous concentration gradient. Hemolytic activity occurs only at the point of optimal concentration ratio [32,300,304]. In early studies, surplus of Hbl B or L1 seemed to hinder this activity [300,323]. On the contrary, current studies show that the hemolytic activity is determined by the amount and the diffusion velocity of Hbl B. Excess of B enhances the outer hemolytic ring, and excess of L1 inhibits it, while the amount of L2 seems not to influence it [301].

What became obvious is that all three components are necessary for biological (hemolytic or cytotoxic) activity, and that a defined concentration ratio is required. More recent studies oppose the theories of a membrane attack complex or the oligomeric Hbl B pore (see above). Using Hbl proteins recombinantly produced in *B. anthracis* and *E. coli*, it was shown that only Hbl B can bind to the surface of target cells. Thus, the current model is that the three components bind to the target cell surface sequentially in the required binding order for pore formation B-L1-L2 [204,301]. Hbl B alone shows neither cytotoxic activity nor pore formation on target cells [204,301] or artificial lipid bilayers [324]. On the other hand, it has been determined that the Hbl components form complexes in solution prior to cell binding, and that Hbl B, recombinant, as well as native in *B. cereus* culture supernatants, is mainly present in those complexes. Recombinant Hbl B binds to L1 with a K_D_ value of 4.7 × 10^−7^ M, and Hbl L2 to L1 with a K_D_ value of 1.5 × 10^−7^ M. Binding of Hbl L2 to B was observed only in a certain constellation and is comparably weak, with a K_D_ value of 3.4 × 10^−6^ M [308]. Furthermore, Hbl B alone was able to form up to 600 kDa multimeric complexes. Those complexes, which still showed pore-forming activity, also comprised Hbl L1 and L2 [324]. Another interesting fact is that heat treatment led to a decrease of pore-forming and cytotoxic activity of Hbl at approximately 60 °C, and a subsequent increase at higher temperatures. The latter is caused by releasing Hbl B monomers from the tight complexation observed earlier, thus enhancing Hbl B binding to the target cell surface [324]. These initially contradictory findings, of only Hbl B binding to the cell surface, and of its tight complexation, can nevertheless be equally appropriate. Similar to the above described ClyA mechanism, homo-(Hbl B) as well as hetero-oligomers (Hbl B-L1 and L1-L2) would be present in solution, letting the final assembly of the pore at the cell surface take place immediately. Rapid Hbl pore formation has been shown [227,301]. However, not only do the binding order and pre-formed complexes play a role in Hbl pore formation, but also to a large extent the concentration ratio of the three components. Most rapid pore formation was detected with ratios L2:L1:B = 1:1:10 and 10:1:10, meaning that surplus of Hbl B enhances, and surplus of Hbl L1 hinders the velocity of pore formation, while the amount of Hbl L2 seems not to be crucial [301]. It has further been proven that binding of Hbl B to the cell surface is the most essential step for pore formation and cytotoxic activity. Most interestingly, this binding step is enhanced in the presence of Hbl L1 [301], similarly to the presence of certain Hbl B-specific monoclonal antibodies, which has been described as antibody-dependent enhancement [308,325]. Again, these findings attribute a major role in Hbl pore formation to the pre-formed B-L1 complexes, which seem to be as necessary as sufficient amounts of free Hbl B. Recently, new insights into the properties of the Hbl pores were gained. Experiments on planar lipid bilayers demonstrated that the Hbl pores have a rather small single channel conductance of around 200 pS and a probable channel diameter of at least 1 nm [324]. This correlates with earlier observations from osmotic protection experiments using carbohydrates, in which the channel diameter was estimated at 1.2 nm [300]. Moreover, the Hbl pores seem to be highly instable and of a limited lifetime, as well as slightly cation selective [324].

**Figure 5:**
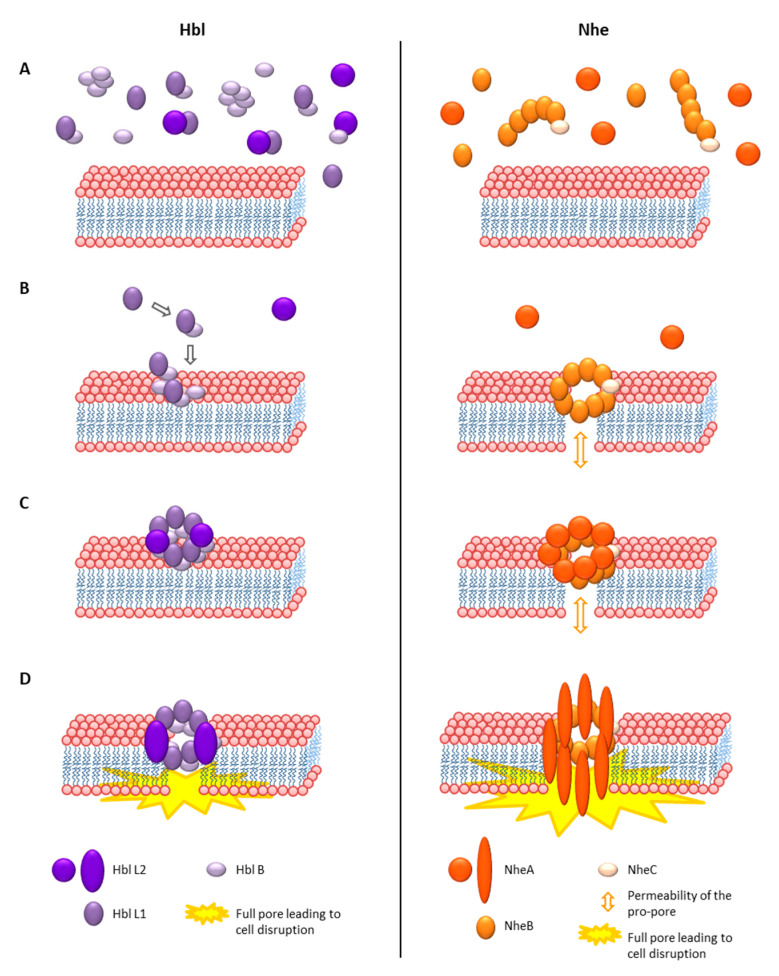
Comparative depiction of the pore forming mechanism of the three-component enterotoxin complexes Hbl and Nhe. (**A**) Step 1: Complex formation in solution as well as presence of free Hbl and Nhe components [301,308,324,327,329,330]. (**B**) Step 2: Membrane attachment as Hbl B-L1 complexes [204,301], or as NheB-C complexes resulting in small, permeable “pro-pores” [327,330]. (**C**) Step 3: Attachment of Hbl L2 or NheA [301,324,329]. (**D**) Step 4: Conformational changes and completion of the full pore [301,324,329].

Detailed investigations of the mode of action of Nhe have also been conducted. Generally, these made faster progress compared to Hbl, as no second enterotoxin complex disturbed the tests, and natural [326,327,328] as well as constructed [217] mutants of the single Nhe components existed. When Nhe was first discovered in the 1990s, no hemolysis on sheep blood agar could be detected, thus it was mistakenly described as “non-hemolytic”. However, Nhe shows hemolytic activity against erythrocytes depending on the species used. It is furthermore cytotoxic towards different epithelial cells, as shown by loss of ATP and LDH [217]. This study initially described Nhe as a pore forming toxin producing large conductance channels, which leads to osmotic swelling and, finally, lysis of the epithelial cells. All three Nhe components are necessary for maximum biological activity *in vitro*, which also requires a specific concentration ratio (NheA:B:C = 10:10:1) [209]. In a more recent study, varying ratios were detected in 12 *B. cereus* isolates, of which some showed a slightly higher (3:1) or a slightly lower (1:2) NheA:B ratio, which did, however, not influence the toxic activity [329]. A specific binding order of the three components is also necessary for pore formation [209]. In the study of Lindbäck et al., only NheB was able to directly bind to the surface of the target cell, and binding was abolished by excess of NheC. On the other hand, it has also been suggested that NheB as well as NheC can bind independently to the target cell surface and that NheC presence is mandatory in the first, and NheA in the final step of pore formation [328]. However, NheB and C form highly affine and stable complexes in solution (K_D_ value of 4.8 × 10^−10^ M [330]), which impedes the detection of NheC in the supernatant of *B. cereus* cultures via specific antibodies. This might be the reason why NheC has initially not been detected as part of the Nhe toxin complex [33,207,306], and why it is generally not found in the *B. cereus* culture secretome or only in very low concentrations [53,226,248]. The approximately 620 kDa NheB-C complexes consist of at least one NheC molecule and up to 15 NheB molecules [330]. Furthermore, the Nhe components seem to be additionally processed in the extracellular space, after separation from the signal peptide for secretion. The shorter forms lack up to 12 additional *N*-terminal amino acids while remaining their cytotoxic activity [33,306,331]. A defined ratio of NheB and C in the complexes (NheB:C = 10:1), as well as a sufficient amount of free NheB, is essential for effective cell binding and the cytotoxic activity of Nhe [327]. Moreover, in an appropriate concentration ratio, the NheB-C complexes themselves are capable of forming stable transmembrane channels with a diameter of approximately 2 nm, and thus increase membrane permeability of the target cells even before formation of the whole pore [330]. To complete the pore, NheA, which is not able to interact with NheB or C in solution, binds to cell-bound NheB. For this, an NheB:C ratio of 10:1 is also mandatory. Artificial addition of NheC resulting in a ratio of NheB:C = 1:1 led to reduced cell binding of NheB [327] as well as to reduced amounts of NheA at the cell surface [329]. Evidence has been found that the *N*-terminus of NheA is important for attaching to cell-bound NheB-C and that NheB is the main interaction partner for NheA [329]. Several studies proved that the pore forming procedure involves massive conformational changes of all protein components and that especially NheA is highly flexible [326,329,332], similarly to ClyA (see above). Osmotic protection and lipid bilayer experiments have also pointed to a comparable size of the ClyA and the Nhe full pores [217]. Another similarity is the permeabilization of the target cell membrane even before formation of the full pore [321,330]. A recent study showed that NheABC (ratio 10:10:1) forms large, stable pores with a conductance of approximately 18 nS on planar lipid bilayers. Increased amounts of NheC led to smaller and instable pores [309]. What all three (ClyA, Nhe and Hbl) pores seem to have in common is their selectiveness for cations [217,315,324]. Distinct features of the ClyA, Hbl and Nhe pores are comparatively summarized in Table 1. Figure 5 illustrates similarities and differences in the mechanism of pore formation of Hbl and Nhe.

To sum up, both enterotoxin complexes need all three components for full biological activity [204,209,301]. In a first step, the components of both toxins form complexes in solution. Hbl B-L1, Hbl L2-L1, and Hbl L2-B form complexes with decreasing affinities [308]. Moreover, Hbl B itself oligomerizes [324]. On the other hand, NheB and C binding is highly affine, while NheA is not able to interact with the other components in solution [329,330]. In both cases, sufficient amounts of free Hbl B and NheB, the binding components, are also required [301,324,327], but complex formation seems to be a great advantage for the efficiency of the formation of both pores. In a second step, the NheB-C complexes attach to the cell membrane and form small, permeable pro-pores [327,330]. In case of Hbl, only Hbl B binds to the cell surface, but binding is enhanced in the presence of L1 [301]. Free Hbl L1 can subsequently bind to B [204,301]. Excess of Hbl L1 as well as NheC can impair the pore forming process. The optimal concentration ratio for maximum pore formation is Hbl L2:L1:B = 1:1:10, and NheA:B:C = 10:10:1 [209,301,327]. The third step consists of attachment of Hbl L2 or NheA. Especially for Hbl L2, only small amounts are needed compared to the other components [301,324,329]. Massive conformational changes, which are proven only for Nhe so far [329], lead to the formation of the full pore and permeabilization of the target cell membrane in the fourth step (see also Figure 5).

Though both are slightly cation selective, the size of the full Hbl and Nhe pores differs. Moreover, the Nhe pores seem to be more stable and persistent on planar lipid bilayers than the Hbl pores (see Table 1). On the other hand, Hbl pore formation seems to be more rapid and efficient [227,301]. In one of our own studies, Hbl-positive strains caused significantly faster influx of propidium iodide into target cells compared to solely Nhe producing isolates. Nevertheless, the latter were responsible for significantly increased cytotoxicity after 24 h [227]. Thus, it can be concluded that Hbl pores are formed more quickly, but that Nhe pores, not least due to their size, cause more damage to the target cell membrane. Once again, this indicates a different mode of action of the two enterotoxin complexes. It has been speculated that Nhe is the more dominant enterotoxin, as it is more frequently present, cytotoxicity correlates well with the amount of NheB, and toxic activity was eliminated by deletion of *nhe* genes [5,53,217]. On the other hand, disruption of the *hbl* genes also resulted in decreased cytotoxicity in a different strain, and residual cytotoxic activity was observed after removal of Nhe from culture supernatants [205,227]. In conclusion, both enterotoxin complexes seem to be (strain-specifically) important for cytotoxicity and thus the course of the diarrheal disease. If unleashed simultaneously, the Hbl pores might be responsible for a rapid, initial damage of the target cell membranes, while the Nhe pores cause delayed but stronger harm.

Despite all the progress made in exploring the mode of action of the tripartite enterotoxins, the exact regions and amino acids responsible for the protein-cell and protein-protein-interactions still remain to be identified. Nevertheless, by employing mutated NheC proteins, it was shown that its hydrophobic β-tongue is essential for cell binding, but not for the interaction with NheB in solution [328]. Based on studies with Nhe-specific, neutralizing monoclonal antibodies (mAbs), first hints were found that the *C*-terminal region of NheC is necessary for interaction with NheB [327,330]. Similarly, it was found that amino acids 122–150 of NheB (binding site of mAb 2B11) are the counterpart essential for the interaction with NheC, and that amino acids 321–341 (epitope of mAb 1E11) are needed for association between NheA and cell-bound NheB [326]. The authors concluded that the *C*-terminus is an important functional region of NheB. Furthermore, amino acids 85–100 of NheA (epitope of mAb 2G11) are relevant for attachment of NheA to the NheB-C complex on the cell surface, and amino acids 130–160 (binding site of mAb 1G4) play an important part in the change of conformation of NheA for transition of the NheABC-complex to the full-pore [329].

A great heterogeneity of enterotoxin production exists among enteropathogenic *B. cereus* isolates. In the supernatants of 100 strains, Moravek et al. found concentrations of 0.03–14.2 µg/mL NheB and 0.02–5.6 µg/mL Hbl L2, while the median NheB level was significantly higher for food poisoning than for food or environmental isolates [53]. A strong correlation between the amount of NheB and cytotoxic activity has been determined [53,227]. Further correlation was found for the amounts of Hbl B and L1 [227]. Defining the effective dose of the enterotoxins proved to be rather difficult. In cytotoxicity tests on Vero cells, Lund and Granum found a 50% inhibitory concentration of 20–30 ng for each Nhe component of strain NVH 0075-95 and 25–30 ng for each Hbl component of strain F837/76 [306]. In supernatants of high toxic strains, NheB amounts of approximately 10–14 µg/mL are found [53,326]. When these supernatants are tested in cellular assays, reciprocal cytotoxicity titers of usually more than 1000 are obtained [226], leading to the assumption that Nhe is toxic even at pmol concentrations. Similarly, propidium iodide influx into Vero cells as a measure of pore formation was maximal at a Hbl concentration of 68.3 pmol/mL (Hbl L2:L1:B = 1:1:1) and still detectable after four hours of measurement at a concentration of 4.7 pmol/mL [301].

Since their discovery, it was believed that the above described three-protein-component enterotoxins are a unique feature of the *B. cereus* group [5]. Just recently, extensive *in silico* search revealed Hbl and Nhe orthologues, and tripartite toxins of the ClyA family, in Gram-negative bacteria such as *Serratia marcescens*, *Erwinia mallotivora* and *Aeromonas hydrophila*. Furthermore, the tripartite α-pore forming toxin AhlABC of *A. hydrophila* was characterized in detail [336]. Briefly, the AhlC component forms a tetramer and subsequently disaggregates into monomers for target membrane binding, which leads to recruitment of AhlB. Major conformational changes initiate assembly of AhlB into a hydrophobic pore. With the subsequent binding of AhlA, the active, hydrophilic-lined pore is formed [336]. After that discovery, however, a further unique characteristic of the *B. cereus* group would be a toxin consisting not only of three, but of four individual, active protein components. As described in Section 4.1.1, the more common *hblCDAB* operon includes a fourth gene encoding Hbl B’, which shows some sequence similarities, but also differences compared to Hbl B. It has been suggested that *hblB* is transcribed independently from *hblCDA* [206]. Consensually, total transcriptome analyses revealed that *hblB* was down-regulated in reference strain F837/76 in the presence of mucin, while *hblA* was up-regulated [278]. Moreover, the protein was found in the secretome of *B. cereus* type strain ATCC 14579 [206]. Our own, recent data bear evidence that the Hbl B’ protein indeed plays a role in the complex formation and pore-forming activity of Hbl and it should thus be considered as the fourth component of the rather quadripartite Hbl enterotoxin complex. Furthermore, we detected it in culture supernatants of several enteropathogenic *B. cereus* strains, which increases its relevance (data unpublished).

#### 4.1.4. Susceptibility and Cellular Response towards the Tripartite Enterotoxins

Several studies proved that the *B. cereus* enterotoxins affect a variety of different target tissues and cell lines, such as rabbit retinal tissue and ileum [303], sheep blood [300], as well as Vero (African green monkey kidney), CHO (Chinese hamster ovary), HUVEC (human umbilical vein/vascular primary endothelial cells), Hep-2 (human cervix), CaCo-2 (human colon), Hep-G2 (human liver), A549 (human lung), RPMI 8226 (human B lymphocyte), A204 (human muscle), Jurkat (human T lymphocyte), U937 (human monocyte), HT-29 (human colon), IPEC-J2 (swine colon), BMDM (primary bone marrow derived macrophages), EC (mouse lung primary endothelial), RAW 264.7 (murine macrophages), B16-BL6 (murine skin), B16-F10 (murine skin), and HT-1080 (human connective tissue) cells [204,227,306,335,337,338]. At this point, the question frequently arose whether specific target structures and receptors for Hbl and Nhe exist. Generally, pore forming toxins get to their target cell membrane in their soluble form by binding to proteinaceous receptors or by specifically interacting with different lipids [339]. The Cry toxins from *B. thuringiensis,* for instance, interact with insect-specific receptors such as cadherin-like proteins, aminopeptidase N, alkaline phosphatase or ATP binding transporters [340,341]. For *S. aureus* hemolysins, specific target proteins such as disintegrin are known, and *C. perfringens* enterotoxin (CPE) attaches to claudin [339]. The protective antigen (PA) from *B. anthracis* binds CMG2 (capillary morphogenesis gene 2 or ANTXR2) and TEM8 (tumor endothelial marker 8 or ANTXR1) receptors at the cell surface. Both are integrin-like proteins associated with proteins of the extracellular matrix, such as collagen and fibronectin [342]. On the other hand, Cyt toxins from *B. thuringiensis* recognize membrane lipids such as phosphatidylcholine, phosphatidylethanolamine or sphingomyelin [107,343]. Actinoporins for instance interact with sphingomyelin, perfringolysin O and listeriolysin O with cholesterol. Cholesterol is also listed as target structure for ClyA, Hbl and Nhe [339], however, this has only been experimentally proven for ClyA (see Table 1 and [316,321,334]). On the one hand, the broad range of target cells (see above), as well as the ability of NheB-C [330], NheABC [309] and Hbl [324] to form pores in artificial lipid bilayers, oppose the existence of specific (protein) receptors. On the other hand, Hbl and Nhe contribute differentially to total cytotoxicity of *B. cereus* supernatants. In a study from 2014, total cytotoxic activity of strain F4430/73 on HUVEC was dominated by Nhe (90%), while on A549 cells 75% of the cytotoxic effects could be attributed to Hbl. Further on, U937 cells proved to be nearly resistant towards Nhe [227]. These variations point to specific enterotoxin target structures. Specific receptors also best fit the proposed models for Hbl and Nhe pore formation and mode of action (Section 4.1.3). Moreover, it is believed that Hbl and Nhe address different receptor types, which would also account for the non-demonstrable interaction between both toxin complexes despite sequence homology [204,227]. A big step towards resolving this long-discussed issue has recently been made. Using a whole-genome CRISPRCas9 knockout screen, LPS-induced TNF-α factor (LITAF) was identified as the main receptor for Hbl. Moreover, its related protein CDIP1 was found to be an alternative Hbl receptor in the absence of LITAF. LITAF/CDIP1 double knockout cells were completely resistant towards Hbl B binding as well as Hbl toxicity, and LITAF knockout mice were resistant towards Hbl toxicity [335]. Nevertheless, the specific Nhe receptor, which is more than likely to exist, is still unidentified.

Further open questions regard the specific cellular response towards the *B. cereus* enterotoxins. Generally, a great variety of cellular reactions to pore-forming toxins exist. First, pore formation decreases the intracellular potassium concentration, which activates different signal pathways including the inflammasome complex (e.g., aerolysin, *Vibrio cholerae* cytolysin, *Clostridium* β-toxin or *S. aureus* toxins), MAPK (mitogen-activated protein kinase) signal pathways or autophagy (e.g., *S. marcescens* ShlA or *S. aureus* α-hemolysin). Pore formation in the cell membrane can also induce calcium influx, which again activates different signal cascades [339,344]. It has further been reported that pore forming toxins alter gene expression in the target cells [345]. When cells are exposed to small toxin concentrations, or over a short period of time, mechanisms for membrane repair become effective, which mostly depend on calcium. Pores can be taken up via endocytosis and further be degraded or emitted. Alternatively, membrane vesicles including the toxins can be separated [339,345]. Liu et al. showed that Nhe is able to induce apoptosis in Vero cells. As mentioned above, the formation of the Nhe pore leads to calcium influx into the cells, oxidative stress, and thus to an activation of ASK1 (apoptosis signal-regulating kinase 1) and p38 MAPK. The latter is additionally activated by Fas (tumor necrosis factor superfamily receptor). The kinases subsequently activate caspase-8 and caspase-3 for induction of apoptosis [346]. Recently, it has been shown that Hbl as well as Nhe is able to induce inflammasome-mediated mortality of macrophages [337,338]. Formation of the lytic pore at the macrophage cell membrane leads to potassium efflux, subsequent activation of the NLRP3 (NOD-, LRR- and pyrin domain-containing protein 3) inflammasome, secretion of interleukin-1β and interleukin-18, and pyroptosis, an inflammatory form of cell death. The authors concluded that Hbl and Nhe operate synergistically to induce inflammation and that the detection of inflammasome-activating toxins in the cytosol is essential for recognizing a *B. cereus* infection [337,338]. Hence, the involvement of *B. cereus* and especially its enterotoxins in gastrointestinal disease and inflammation is ensured.

Beyond that, *B. cereus* is responsible for a variety of severe extra-gastrointestinal infections. Often neonates, elderly or immunosuppressed patients are affected [347,348], but infections of immunocompetent persons are also known [349]. These include local, especially post-operative skin and wound infections [347,349,350], septicemia [350,351,352,353,354,355,356,357,358,359,360], meningitis [361,362,363,364,365,366,367,368,369,370,371,372], pneumonia (often by *B. cereus* strains exhibiting *B. anthracis* toxin genes) [373,374,375,376,377,378] and endocarditis [379,380,381,382,383,384,385,386,387,388]. Several studies focus on *B. cereus*-associated endophthalmitis, which occurs mainly following post-traumatic injuries and often leads to loss of vision or even of the eye in less than 48 h. Severe inflammation is caused by the concerted action of rapid bacterial replication, migration and toxin production [389]. Early studies found evidence for an involvement of Hbl, phosphatidylcholine-preferring phospholipase C and collagenase in *B. cereus* endophthalmitis [302,303]. By now, it is believed that the combination of multiple virulence factors and toxins leads to intraocular virulence [389]. Using deletion mutants, it was shown that the quorum sensing regulator PlcR (see also Section 4.1.2) contributes significantly to the disease, on the one hand by controlling toxin and virulence factor expression, but also by controlling expression of cell wall components and bacterial motility [287,390,391]. Motility and swarming behavior, as well as cell wall components such as flagella and pili or the S-layer are also a trigger for intraocular inflammation [392,393,394,395]. The disease results from the interplay of various bacterial and host factors [389,396,397], which makes clinical treatment difficult. Applying sublethal doses of carvacrol, for instance, enhanced *nhe* and *hbl* gene expression and toxin production, as well as stress-induced virulence against *Caenorhabditis elegans* and mice [398]. Further recent studies focus on genotyping, whole genome sequencing and, thus, on the identification of other putative *B. cereus* virulence factors involved in endophthalmitis [399,400,401]. The most important, which may also play a role in gastrointestinal diseases, are summarized in Section 5.

### 4.2. Cytotoxin K

In contrast to the above described tripartite toxin complexes Hbl and Nhe, the third diarrheal enterotoxin, cytotoxin K, is a single protein, β-barrel pore-forming toxin. CytK was first isolated from thermotolerant *B. cereus* strain NVH 391/98, which was responsible for a severe food poisoning case in a nursing home in France in 1998. Six people suffered from bloody diarrhea, and three people died. At this time, none of the known *B. cereus* enterotoxins could be identified, and CytK proved to be toxic towards Vero cells [31]. Nevertheless, Nhe was identified later in this strain [211,215], which might also have been involved in the outbreak. Concurrently, CytK was also isolated from supernatant of a *B. cereus* strain associated with endophthalmitis and named hemolysin IV (HlyIV) [302].

The CytK toxin is encoded by the single gene *cytK*. First PCR analyses suggested that *cytK* is not particularly prevalent among different *B. cereus* isolates [31,402]. More detailed investigations identified two different variants of the *cytK* gene with high sequence homology, which were—according to their discovery—named *cytK-1* and *cytK-2*, respectively [403,404]. The corresponding toxins CytK-1 and CytK-2 share 89% amino acid sequence identity. While *cytK-2* is found in a variety of *B. cereus* strains, the *cytK-1* variant occurs only exceptionally. Among the *B. cereus* group, *cytK-2* was most commonly found in the mesophilic phylogenetic groups III and IV [145,404]. In a study from 2015, the occurrence of *cytK-2* was lower in clinical and food poisoning isolates compared to environmental strains, which allowed the authors to conclude that CytK-2 is most likely not a relevant virulence factor for the diarrheal syndrome [405]. In our own studies, we also concluded that *cytK-(2)* is not a suitable marker for enteropathogenic *B. cereus* or their cytotoxic activity [91,297]. Basically, *cytK-1-*positive strains bear an unusual variant of the *nhe* operon [215]. For a long time, only five of these strains were known [406], which were eventually defined as a new species, *B. cytotoxicus.* Their 16S rRNA gene sequences show a close phylogenetic relationship to other members of the *B. cereus* group, but within this group the investigated *B. cytotoxicus* strains form their own cluster [95,404]. In more recent studies, *B. cytotoxicus* was identified with higher prevalence. Of 151 tested potato products, 35% were positive, with highest prevalence in dried potato powder [407]. Nine out of nine mashed potato powders were positive for *B. cytotoxicus*, which, however, showed highly variable cytotoxic activity [408]. The species was also identified in millet flour and potato-containing products, especially in potato flakes [409]. When the genome sequences of 14 *B. cytotoxicus* strains were compared, they could clearly be distinguished from other members of the *B. cereus* group, and a hydroxy-phenylalanine operon for utilization of tyrosine was identified as a unique feature for this species. The authors concluded that this putative evolutionary advantage might have been the reason for species differentiation [410]. Recently, Cavello et al. first reported the isolation of *B. cytotoxicus* from the natural environment, a geothermal area, as well as its keratinolytic activity [411]. Interestingly, *cytK-2* is not present in *B. anthracis* [125], but has been found in *B. mycoides* [412], very frequently in *B. thuringiensis* [125,210], and also outside the *B. cereus* group in *Paenibacillus cookie* [413].

**Figure 6:**
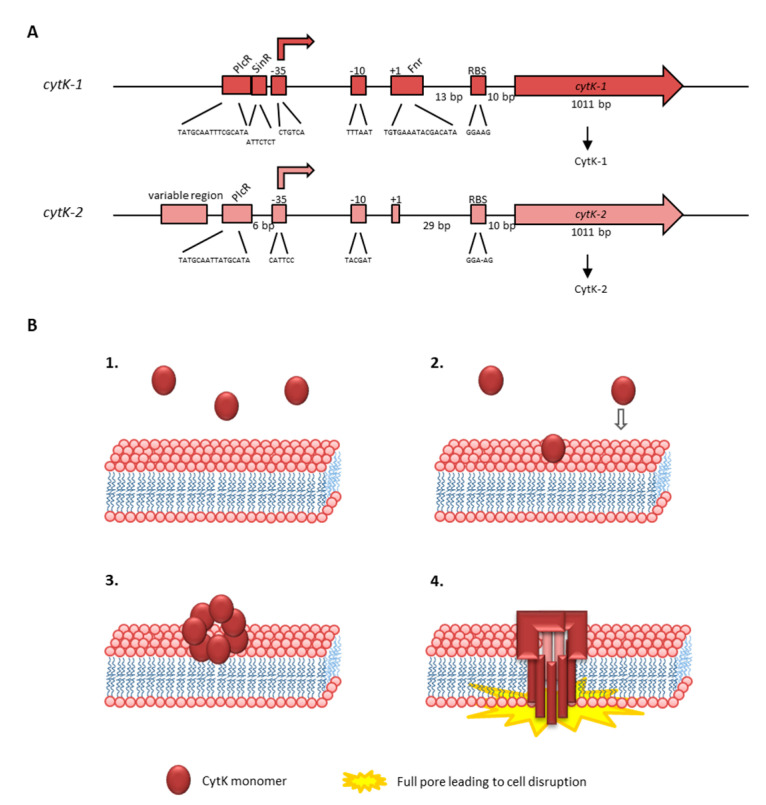
Properties of the CytK enterotoxin from *B. cereus*. (**A**) The *cytK-1* and *cytK-2* genetic regions according to Böhm et al. [210]. The promoter regions including (putative) transcriptional regulator binding sites are shown. (**B**) Putative mode of action of CytK during pore formation according to related β-barrel pore-forming toxins [419,423,424]. Step 1: Soluble monomers in solution. Step 2: Target membrane binding due to putative interaction with liposomes. Step 3: Oligomerization into heptamers. Step 4: Conformational changes and pore formation.

Like the *hbl* and *nhe* operons, the expression of the genes *cytK-1* and *cytK-2* is regulated by PlcR (compare Section 4.1.2) via binding to specific PlcR boxes in their promoter regions [31,169,246,414,415]. On the other hand, the unusually long 5′ untranslated regions, which were found upstream of *hbl* and *nhe* (compare Section 4.1.1 and 4.1.2), are not present. With approximately 100 bp, the promoter regions are comparably short. Analyzing the sequences of a variety of *B. cereus* group strains, Böhm et al. found high similarity in the *cytK-1* promoter region, and a highly variable region as well as a strongly conserved promoter for *cytK-2* [210]. Figure 6A gives an overview of the genetic organization of *cytK-1* and *cytK-2*. These in silico analyses further revealed putative binding sites for Fnr and SinR (compare Section 4.1.2) upstream of *cytK-1*, which are, however, not experimentally verified to date. It has also been shown that both *cytK* genes are expressed independently of CcpA-mediated catabolite control [233] and that CodY does not interact with their promoter regions [210]. Furthermore, evidence has been found that the *cytK* genes, similarly to *hbl*, are inherited via horizontal transfer as well as frequent deletion [210]. In contrast to the highly conserved *cytK-1* promoter region, great variability in CytK-1 production and cytotoxicity has been observed between the highly toxic type-strain NVH 391/98 and non-toxic NVH 883/00 [215,414], which might be explained by one mismatch in the PlcR binding site of strain NVH 391/98 or by further, as yet unidentified regulatory pathways [215,406]. Another study revealed that only a small subpopulation (1–2%) of *B. cereus* ATCC 14579 expressed *cytK* and produced the toxin [416], pointing again to a yet unknown regulatory mechanism.

Like the Hbl and Nhe proteins, both CytK toxins harbor Sec-type signal peptides, which proposes secretion through the Sec translocation pathway. This was proven by treatment with sodium azide, an inhibitor of SecA, and the subsequent failure of secretion and intracellular accumulation of CytK [283]. On the other hand, no evidence was found for an involvement of the flagellar export system in CytK secretion [288].

The CytK-1 protein initially isolated from strain NVH 391/98 had a size of 34 kDa, and was shown to be hemolytic, dermonecrotic, able to form pores in lipid layers, and highly toxic towards human intestinal epithelial cells [403,417]. The pores were slightly anion selective with a predicted minimum pore diameter of approximately seven Å. Furthermore, the spontaneous formation of SDS-resistant oligomers was reported [417]. CytK-2 also proved to be hemolytic, cytotoxic and pore-forming in lipid layers, but with an approximately 80% reduced cytotoxic activity compared to CytK-1. Additionally, the pores showed lower conductance. The authors further assigned the differences between CytK-1 and CytK-2 to certain regions of the proteins [403]. Nonetheless, considerable amino acid sequence homologies were found to *S. aureus* leukocidin, *S. aureus* α- and γ-hemolysin [418,419], *C. perfringens* β-toxin [31,420,421], and *B. cereus* hemolysin II [406,422]. Thus, CytK is classified as member of the family of β-barrel pore-forming toxins, and, although not yet experimentally verified, its mechanism of oligomerization and pore formation can be predicted from the closely related and well investigated *S. aureus* α-hemolysin and *B. cereus* hemolysin II. Figure 6B shows a model of the predicted mode of action in the style of Hbl and Nhe. Initially, the toxin is secreted as soluble monomer. Those monomers bind to the target cell membrane presumably due to their interaction with liposomes. The subsequent formation of SDS-, but not heat-resistant, oligomers is characteristic for β-barrel pore-forming toxins. Most likely, heptamers are formed and conformational changes occur to complete the mushroom-shaped, anion selective, β-barrel transmembrane pore with hydrophobic parts facing the lipids and hydrophilic parts facing the inside of the channel [322,419,423].

### 4.3. Methods for Detection of the Enterotoxins

In routine food or clinical diagnostics, presumptive *B. cereus s. l.* strains are usually detected on selective culture media according to international standards (ISO 7932:2005-03), highlighting the importance of toxin detection rather than species differentiation [93]. Various PCR, real-time PCR and multiplex PCR systems have been established and used for the detection of the *hbl*, *nhe*, *cytK* and *ces* genes ([48,52,70,114,185,186,187,404,412,425,426,427,428] and many others as summarized in Section 2). With progress in technology, it is also imaginable that these PCR-based techniques will be replaced by other methods such as whole genome sequencing, DNA microarray-based analysis, or fingerprinting techniques [41,54,93,120,143,210,429,430,431,432,433,434,435]. However, the detection of enterotoxin genes or patterns is not a reliable tool to predict the toxic potential of an isolate. This is not only defined by genetic prerequisites, but rather by the number of secreted enterotoxins, which can be highly variable even among strains bearing identical toxin genes [53,91,226,227].

Tools for the detection of the secreted enterotoxins in *B. cereus* culture supernatants are mainly based on monoclonal antibodies against the single toxin components. Three test kits were commercially available, which are Duopath^®^ Cereus Enterotoxins (Merck) detecting NheB and Hbl L2, Oxoid™ BCET-RPLA Toxin Detection Kit specific for Hbl L2, and 3M™ Tecra™ Bacillus Diarrheal Enterotoxin (BDE) Visual Immunoassay (VIA) detecting NheA and NheB [428,436,437,438,439,440,441,442,443,444]. The latter seems to be discontinued. However, of these toxin components, only the amount of secreted NheB strongly correlates with cytotoxicity [53,227]. Using further, antibody-based systems such as sandwich enzyme immunoassays or Western and colony immunoblots, all single Hbl and Nhe components can be reliably detected and quantified [53,226,308,427,445,446,447]. However, it must be noted that these assays do not specifically detect biologically active toxins. At the moment, there is no commercially available detection kit for CytK, but the toxin has previously been detected via specific antisera [215]. As an alternative method, a MALDI-ToF MS system was established for the detection of secreted CytK-1 and Nhe [448], as well as for the differentiation of emetic and non-emetic strains [189,190].

To assess the toxic activity of the *B. cereus* enterotoxins, *in vivo* animal experiments were initially performed, such as the rabbit ileal loop, the guinea pig skin reaction, and the vascular permeability test [35,449,450], as well as monkey feeding experiments [451]. At present, these animal models are widely replaced by cytotoxicity assays carried out in cell culture [33,43,71,134,141,227,417,439,445,452,453,454,455,456,457]. In WST-1 bioassays, for instance, 50% lethal doses are determined and shown as reciprocal titers, which can easily be compared between different isolates [91,227]. Measuring the influx of fluorescent markers such as propidium iodide into the cells allows conclusions on the velocity of pore formation by the enterotoxins [217,227]. From these *in vitro* tests, however, only limited conclusions can be drawn regarding the actual toxicity *in vivo*. This means that it is hard to predict if a certain isolate is capable of causing an infection once consumed, especially as only enterotoxins produced by viable *B. cereus* in the intestine contribute to the diarrheal disease. Thus, many additional factors regarding the host have to be taken into account, which we recently investigated and summarized [27,92,278,297].

## 5. Further Virulence Factors and Toxins

Next to the three enterotoxins, the involvement of further secreted virulence factors in the diarrheal disease is extensively discussed in the literature. The 45 kDa EntFM was first described as enterotoxin in 1991 [455]. Its corresponding genes exist in *B. cereus* as well as in *B. thuringiensis* [458]. In a wide study with 616 isolates, *entFM* was found in all tested strains similarly to *nheABC* [122]. EntFM was described as cell wall peptidase involved in adhesion, motility, and biofilm formation, as well as virulence and cytotoxicity of *B. cereus* [459,460]. The prevalence of *entFM* was assessed in several studies characterizing *B. cereus* group isolates from food or environmental samples (see Section 2.1).

Moreover, four different types of hemolysins are known in *B. cereus*. Hemolysin I or cereolysin O is a member of the family of cholesterol-dependent cytolysins, as for example listeriolysin O [406]. The hemolytic activity of this toxin was first described in 1983 [461]. The 42 kDa hemolysin II belongs, like CytK, which is also described as hemolysin IV, to the family of β-barrel pore forming toxins (see Section 4.2 and [406,422]). HlyII is able to induce apoptosis in macrophages [462]. Interestingly, the corresponding gene *hlyII* is not—unlike most described virulence factors [169,248]—regulated by PlcR, but negatively by the TetR-like transcriptional regulator HlyIIR [463,464]. The *hlyII* gene is additionally regulated by the ferric uptake regulator Fur, and deletion of *fur* reduced virulence of *B. cereus* in an insect model [465,466]. The *hlyII* gene has been shown to appear more often in *B. thuringiensis* than in *B. cereus* [467], and the structure and properties of its β-barrel pore have been elucidated [423,424,468,469,470]. HlyII has so far not been described as cause of the diarrheal syndrome, as it might be inactivated by trypsin in the small intestine [5]. The 24 kDa hemolysin III is also a pore forming hemolysin [471,472], but so far it has not been proven to be secreted by *B. cereus*. Thus, its involvement in pathogenicity remains speculative [406]. Cadot et al. suggested HlyII as well as the two metalloproteases InhA1 and NprA as potential markers for differentiation between virulent and non-virulent *B. cereus* strains, as *hlyII* appeared in their study only in virulent strains, and the expression of *inhA1* and *nprA* was clearly increased compared to non-virulent strains [473].

The metalloproteases InhA1, InhA2 and InhA3 were identified as important virulence factors in an insect model; InhA1, furthermore, seemed to counteract macrophages [474,475,476]. *B. cereus* spores are able to survive inside and escape macrophages. It has been shown recently that the most important effector for this is the mature NprA protein, which is cleaved by InhA1 [477]. Additionally, the phosphatidylinositol- and phosphocholine-specific phospholipase C (PI-PLC and PC-PLC) as well as the collagenase ColA are considered as pathogenicity factors [105,110,478,479,480,481]. The bifunctional protein CalY, formerly known as camelysin, has been shown to play an important role in cell surface adhesion and virulence, as well as in biofilms [482,483,484,485]. Proteome analyses identified the exoprotein EntD as a further important virulence-associated factor for *B. cereus*, and an *entD* deletion strain showed reduced growth, motility and cytotoxicity, suggesting that the protein is strongly involved in motility and toxin production [486]. In this context it is important to note that direct involvement of these virulence factors in the diarrheal form of disease has so far not been verified. In one of our own previous studies, no cytotoxic activity of *B. cereus* culture supernatants could be detected after removal of NheB, Hbl L2 and Hbl B via immunoaffinity chromatography [227]. Only for sphingomyelinase (SMase) could a direct impact on *in vitro* cytotoxicity be shown. *B. cereus* ∆*sph* and ∆*nheBC* seemed to complement each other for maximum virulence [487]. Interaction of SMase with Hbl has also been seen in an earlier study [323]. Similarly, SMase causes lysis of erythrocytes synergistically with PC-PLC as cereolysin AB complex [406]. It was further described as an important virulence factor in sepsis and connected to a reduction of phagocytosis [488,489,490]. In a recent study, we showed that NheB and SMase expression, together with exoprotease activity, correlates well with a complex virulence analysis scheme and might serve as template for fast and easy risk assessment in routine diagnostics [297].

## 6. Conclusions

The processes leading to the diarrheal syndrome caused by enteropathogenic *B. cereus,* as well as the physiological procedures involved in the emetic syndrome caused by the cereulide toxins, are highly complex and—despite extensive research, which is summarized here—still not completely understood. Massive progress has been made investigating the mode of action of the three-component enterotoxin complexes Hbl and Nhe, and first insights into the mechanism of the emetic toxin cereulide have been gained. Nevertheless, there are still many open questions regarding *B. cereus* diarrheal and emetic toxins. For instance, we are just at the beginning of understanding the interplay of the single enterotoxin components and the basic mode of action of cereulide. Moreover, a specific receptor structure for Nhe, or the involvement of the putative fourth component of Hbl, Hbl B’, need to be explored, and information on the exact molecular targets for cereulide and isocereulides are still very limited. Besides, *B. cereus* secretes an entire battery of (putative) further virulence factors, which possibly contribute to pathogenicity, either by interacting directly with the enterotoxins or the epithelia or by influencing further stages of the disease, such as adhesion, immune escape, etc.

## Figures and Tables

**Table 1:** Distinct characteristics of the pores formed by ClyA, Hbl and Nhe.

	ClyA	Hbl	Nhe
**First Identified as Pore Forming Toxin**	1995 [333]	1997 [300]	2008 [217]
**Size of the Monomers**	34 kDa [315]	Hbl L2: 45 kDa; Hbl L1: 36 kDa; Hbl B: 35 kDa; and variable homologues [32,54,213,214]	NheA: 41 kDa; NheB: 39.8 kDa; NheC: 36.5 kDa [207]
**Protein Structure**	Tail domain (5 α-helices) and head domain (hydrophobic β-tongue) [317]	Hbl B: Tail domain (5 α-helices) and head domain (hydrophobic β-tongue) [311]	NheA: Tail domain (shortened *N*-terminal α-helix) and enlarged head domain (amphipathic β-tongue) [219]
**Regulation of Gene Expression**	H-NS, SlyA, Fnr [314]	PlcR, CodY, ResD, Fnr, CcpA, SinR (compare Section 4.1.2)	PlcR, CodY, ResD, Fnr, CcpA, SinR (compare Section 4.1.2)
**Complexes in Solution**	Linear oligomers of different sizes; pairs [313,319,320]	Hbl B-L1; Hbl L2-L1; (Hbl L2-B) [308,324]	NheB-C [327,330]
**Formation of Smaller “Pro-pores”**	Membrane permeabilization for 400 Da molecules [321]	no	2 nm NheB-C „pro-pores“ [330]
**„Receptor“ Binding**	Cholesterol [316,334]	LPS-induced TNF-α factor (LITAF) and CDIP1 [335]	unknown
**Pore Formation as**	Homo-dodecamer [319]	Hetero-oligomer [204,300,301]	Hetero-oligomer [204,209,217,326,329]
**Conformational Changes During Pore Formation**	Yes [313,319]	Assumed	Yes [326,327,329]
**Diameter of the Full Pore**	2.5–3 nm, approximately 10 nS conductance [315]	Approximately 1.2 nm [300,324], approximately 200 pS conductance [324]	>2.8 nm [217], approximately 18 nS conductance [309]
**Stability on Planar Lipid Bilayers**	High [315]	Low [324]	High (NheB-C pro-pore [330] and 10:10:1 full pore [309])
**Selectivity**	Moderately cation selective [315]	Moderately cation selective [324]	Moderately cation selective [217]

## Data Availability

Not applicable.
